# The effects of school-based hygiene intervention programme: Systematic review and meta-analysis

**DOI:** 10.1371/journal.pone.0308390

**Published:** 2024-10-08

**Authors:** Sophia Rasheeqa Ismail, Ranina Radzi, Puteri Sofia Nadira Megat Kamaruddin, Ezarul Faradianna Lokman, Han Yin Lim, Nusaibah Abdul Rahim, Hui Yin Yow, Daarshini Arumugam, Alex Ngu, Annie Ching Yi Low, Eng Hwa Wong, Sapna Patil, Priya Madhavan, Ruslin Bin Nordin, Esther van der Werf, Nai Ming Lai

**Affiliations:** 1 Nutrition, Metabolic and Cardiovascular Research Centre, Institute for Medical Research, National Institutes of Health, Ministry of Health Malaysia, Shah Alam, Selangor, Malaysia; 2 Faculty of Pharmacy, University of Malaya, Kuala Lumpur, Malaysia; 3 Ministry of Health, Malaysia; 4 School of Medicine, Faculty of Health and Medical Sciences, Taylor’s University, Subang Jaya, Selangor, Malaysia; 5 Faculty of Medicine, Bioscience and Nursing, MAHSA University, Jenjarom, Selangor, Malaysia; 6 Homeopathy Research Institute, London, United Kingdom; Xiamen University - Malaysia Campus: Xiamen University - Malaysia, MALAYSIA

## Abstract

Children are susceptible to infections due to frequent participation in school group activities and their often-suboptimal hygiene practices. Frequent infections in children affect school attendance, academic performances, and general health. The effectiveness of school-based hygiene-related intervention programmes need to be informed by updated high-quality synthesised evidence. In this systematic review, we searched PubMed and Cochrane CENTRAL for randomised and non-randomised interventional studies that evaluated school-based hygiene-related interventions. We assessed risk-of-bias (Cochrane risk-of-bias 2 tool), performed random-effect meta-analysis (RevMan 5.4) and rated certainty-of-evidence (GRADE). Thirty-nine trials (41 reports), published from 2011 to 2024 from 22 countries were included. Twenty-three studies contributed data for meta-analysis. All school-based interventions were compared with standard curriculum and demonstrated very low to low certainty-of-evidence due to study methodological limitations and imprecision. Hand-body hygiene interventions may improve knowledge, attitudes and practices (SMD 2.30, 95%CI 1.17 to 3.44, 6 studies, 7301 participants), increase handwashing practices (RR 1.75, 95%CI 1.41 to 2.17, 5 studies, 5479 participants), and reduce infection-related absenteeism (RR 0.74, 95%CI 0.66 to 0.83, 5 studies, 1017852 observations). Genital hygiene interventions may improve attitude (SMD 6.53, 95%CI 2.40 to 10.66, 2 studies, 2644 participants) and practices (RR 2.44, 95%CI 1.28 to 4.68, 1 study, 1201 participants). However, intervention effects on oral hygiene appeared mixed, with worsening of the oral hygiene score (SMD 3.12, 95%CI 1.87 to 4.37, 2 studies, 652 participants) but improved dental hygiene (SMD -0.33, 95%CI -0.53 to -0.13, 3 studies, 4824 participants) and dental caries scores (SMD -0.34, 95%CI -0.52 to -0.16, 4 studies, 2352 participants). Limited evidence suggests that interventions targeting hand-body and genital hygiene practices may improve knowledge, practices, and infection-related absenteeism. However, the effects on oral hygiene intervention appeared mixed. Future research should strengthen randomisation and intervention documentation, and evaluate hygiene-related behaviour, academic performances and health outcomes.

## Introduction

Personal hygiene is essential for maintaining optimal health and preventing the spread of diseases. It encompasses a range of practices, including daily body cleansing, regular tooth brushing, handwashing with soap, nail grooming, wearing clean attire, and adhering to proper cough and sneeze etiquette [1]. Young children often exhibit suboptimal hygiene habits, making them more susceptible to communicable diseases [2–5]. Global prevalence of respiratory infections and diarrhoea remains a significant concern, with the United Nations Children’s Fund (UNICEF) identifying them as leading causes of mortality in children under the age of five, accounting for over 2 million deaths worldwide annually [6]. Infectious diseases in childhood often lead to increased school absenteeism, poor academic performance and health status [7–10]. Specifically, childhood diarrhoea has been linked to long-term cognitive deficits [11].

The Coronavirus Disease 2019 (COVID-19) pandemic spotlighted schools as potential disease transmission hubs, partly due to students’ inadequate knowledge and personal hygiene practices [12]. School-to-home transmission of COVID-19 increased containment challenges, leading to suboptimal disease management [13] and an upsurge in reported antibiotic resistance, particularly in low- and middle-income countries [14–16]. However, the pandemic has also reignited interests in the role of hand hygiene in disease prevention. For example, Cambodia reported a 20% increase in the number of group handwashing facilities, with secondary schools witnessing the most significant improvements [17]. On the other hand, nationwide surveys in Ecuador and Sudan in 2020 and 2021 respectively raised concerns that only half of the schools had basic hygiene services or facilities, with only a minority in satisfactory condition [18].

School-aged children dedicate a substantial portion of their time to group activities in school, and this poses a risk of communicable diseases transmission. Multifaceted approaches in national-level hand hygiene campaigns across Europe have been observed, however, these campaigns had a particular emphasis on healthcare professionals [19].

There is a relative lack of emphasis on education concerning daily hygiene practices at home and at school. Global and national infection control and antimicrobial resistance (AMR) strategies have traditionally focussed primarily on healthcare facilities rather than schools [20, 21]. It is imperative to instil comprehensive awareness and knowledge of hygiene practices, including judicious antibiotic use, among students through dedicated health education programs integrated into the school curriculum, with active involvement of educators and designated healthcare personnel. Such preventive strategy is likely more effective than the use of antibiotics in treating infections when they occur. Educational resources, such as the e-bug campaign operated by the UK Health Security Agency, provide free materials for schools to educate students on hygiene, infections, and antibiotics [22].

In addition to health education, the availability of essential hygiene facilities, such as handwashing stations with clean water, is vital for promoting and sustaining good hygiene practices. The WHO/UNICEF Joint Monitoring Programme (JMP) underscores the necessity to extend water, sanitation, and hygiene (WASH) facilities beyond households, with the initiation of WASH in Schools (WinS) project, which has been piloted in countries such as Belize, Guatemala, Honduras, India, Indonesia, the Philippines and Kenya [23, 24]. The projects focus on building capacity in training teachers to incorporate hygiene programming into classroom lessons and school amenities. Examples included using the school’s sanitation facilities as a site for interactive and integrated practical learning, for example, supervised handwashing sessions with soap before eating and after using the toilet [18]. The intended result is that children acquire knowledge about disease transmission and skills in good hygiene practice, which they can take home to their families [23, 24]. Studies have shown that the availability of handwashing facilities is associated with reduced reported illness and absenteeism and increased hygiene knowledge among school children [18, 25, 26].

Mass education in promoting personal hygiene awareness and practices is widely considered essential in safeguarding the health of the younger population, although the extent of its effectiveness in improving knowledge and practice among different settings, evaluated through rigorous studies, remain unknown. We aimed to synthesise the effectiveness of school-based hygiene-related intervention programmes, specifically to look into its impact on improving knowledge, practice and reducing infection-related morbidities.

## Methodology

The systematic review is registered with The International Prospective Register of Systematic Reviews (PROSPERO) (CRD42021253638).

### Eligibility criteria

We included randomised and non-randomised controlled trials, cluster randomised controlled trials, and crossover trials. Studies conducted in preschool, primary school, secondary school, and general college students were included. Studies conducted in healthcare-related colleges or among healthcare students (such as student nurses and dental nurses) were excluded as changes in outcomes do not represent that of the general population.

We included any form of intervention (educational, behavioural, and structural) implemented to improve hygiene-related outcomes, compared with no intervention or any other form of intervention that aimed to improve hygiene-related outcomes, as detailed below.

### Outcome measures

1. Level of knowledge on personal hygiene including characteristics and function of hand hygiene, body hygiene, oral hygiene, and menstrual/genital hygiene. We accepted any tools used by the authors of the individual studies that covered awareness, knowledge, and practices of hygiene in combinations or separately.
2. School absenteeism due to infections or sickness, in particular communicable diseases. We accepted medically documented and self-reported causes of school absenteeism as well as repeated reports for the same student. The types of infections included respiratory infections (characterised by symptoms such as sore throats, coughs, asthma, and influenza-like illness), gastrointestinal infections (characterised by symptoms such as diarrhoea and vomiting), and unspecified infections requiring prescription of antibiotics or other treatments.
3. Changes in oral hygiene status using standard measurement tools such as the Oral Health Index Simplified (OHI-S) score, plaque score, modified gingival index (MGI), and others.
4. Changes in dental status using standard measurement tools such as Decayed-, Missing-, Filled Teeth (DMFT), Decayed-, Missing-, Filled Surfaces (DFMS), and others.
5. Changes in dental caries using standard measurement tools such as International Caries Detection and Assessment system (ICDAS II) and others.

### Search strategies

We searched for relevant studies in MEDLINE (PubMed), Cochrane Central Register of Controlled Trials (CENTRAL) (which covers EMBASE and CINAHL for published studies, and trial registries including the WHO International Trial Registry Platform and ClinicalTrials.gov for ongoing studies). Latest database search was performed on 16^th^ April 2024. No language and publication period restrictions were applied. Relevant clinical trials and conference abstracts were reviewed and searched. The search strategy is summarised in S1 Appendix.

### Selection of studies, data extraction and management

**Fig 1:**
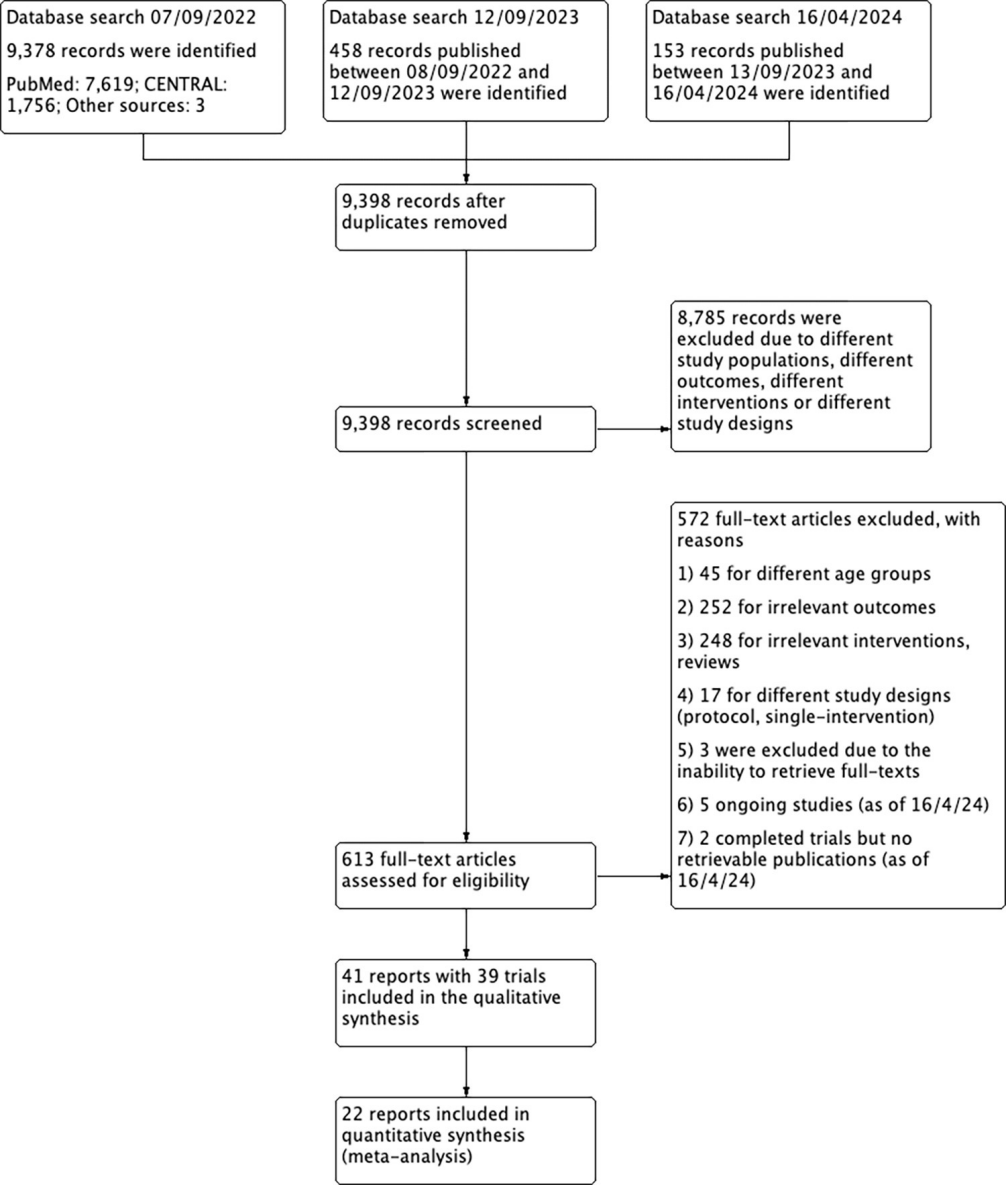
PRISMA flowchart of included studies.

Titles and/or abstracts were independently screened in duplicates by three pairs of authors (SRI & RAR, LHY & AN, ACYL & DA) for potentially eligible studies. Four authors (SRI, RAR, LHY & EFL) subsequently evaluated the shortlisted abstracts and full texts to determine eligibility. We outlined our study selection process in a PRISMA diagram (Fig 1). Three authors (RAR, LHY & EFL) independently extracted all data from each included study using a standardised data collection form which included study design, study population, intervention details, comparison details, and outcomes. All extracted data were evaluated by SRI, EFL and PSNMK for accuracy.

### Risk of bias assessment

The Revised Cochrane risk-of-bias tool for randomised trials (RoB 2) was used to assess the risk of bias in individually randomised, parallel-group trials with regards to the results of the main outcomes. There are five domains explored in this RoB 2 tool: risk of bias arising from the randomisation process (Domain 1), risk of bias due to deviations from the intended interventions (Domain 2), risk of bias due to missing outcome data (Domain 3), risk of bias in the measurement of the outcome (Domain 4), and risk of bias in the selection of the reported result (Domain 5). Within each domain, there are a series of signalling questions with multiple response options (Yes/ Probably Yes/ Probably No/ No/ No Information) to elicit information that is relevant to the assessed domain. Additional considerations arising from period and carryover effects were recommended for crossover trials [27]. The Revised Cochrane risk-of-bias tool for cluster-randomised trials (RoB 2 CRT) was used to assess the risk of bias in cluster-randomized trials. This tool contains six domains. In addition to the five domains in RoB 2, Domain 1b assesses the risk of bias arising from the timing of identification or recruitment of participants [27]. Two review authors (SRI and RAR) assessed each included trial. Each domain was judged as ‘Low’, ‘Some concerns’, or ‘High’ risk of bias based on the answers to the signalling questions algorithm. In general, studies were judged as ’Low Risk of Bias’ if they met the criteria for low risk in all domains, ’Some Concerns’ if there were concerns in at least one domain, and ’High Risk of Bias’ if there was high risk in at least one domain or multiple domains with some concerns. The overall summary of the risk of bias assessment of included studies is reported in S3 Appendix.

### Data synthesis

Outcomes were extracted and included in the quantitative analysis for the same comparisons. For the knowledge, attitudes, and practices of hand hygiene outcomes, all measurement tools with the same outcome were included. For studies that did not report standard deviations (SD), the SD were calculated from the reported confidence intervals (CI) or from given quantile values. The denominator in the measurements for handwashing with and without soap practices was the total number of observed target events. The denominator used in the school absenteeism measurements was the total number of possible absent days/weeks.

For outcomes measured by questionnaires or scores, mean differences (MD) of the total points after intervention were used as the summary measure. For event-based outcomes, risk ratios (RR) were used as the summary measure. MD, RR, and corresponding 95% CI were calculated based on the extracted values and denominators described earlier, with the RR being the ratio of the intervention over control or MD being the difference in total score between the intervention and control. Results were pooled using random effects meta-analysis using the Mantel-Haenszel method for analysis to accommodate the differences across the studies (RevMan 5.4). Absolute risk values were calculated with GRADEpro GDT software. Risk of bias, inconsistency, indirectness, imprecision, and publication bias were used for the GRADE considerations. We justified all decisions to downgrade the certainty of evidence in the footnotes. To further explore the differences across different comparisons, we performed several subgroup analyses. In hand hygiene interventions, we explored the relative effect of studies which had interventions lasting more than one month. In oral hygiene intervention comparisons, we explored the relative effect of studies conducted in different settings.

## Results

### Study selection

**Fig 2:**
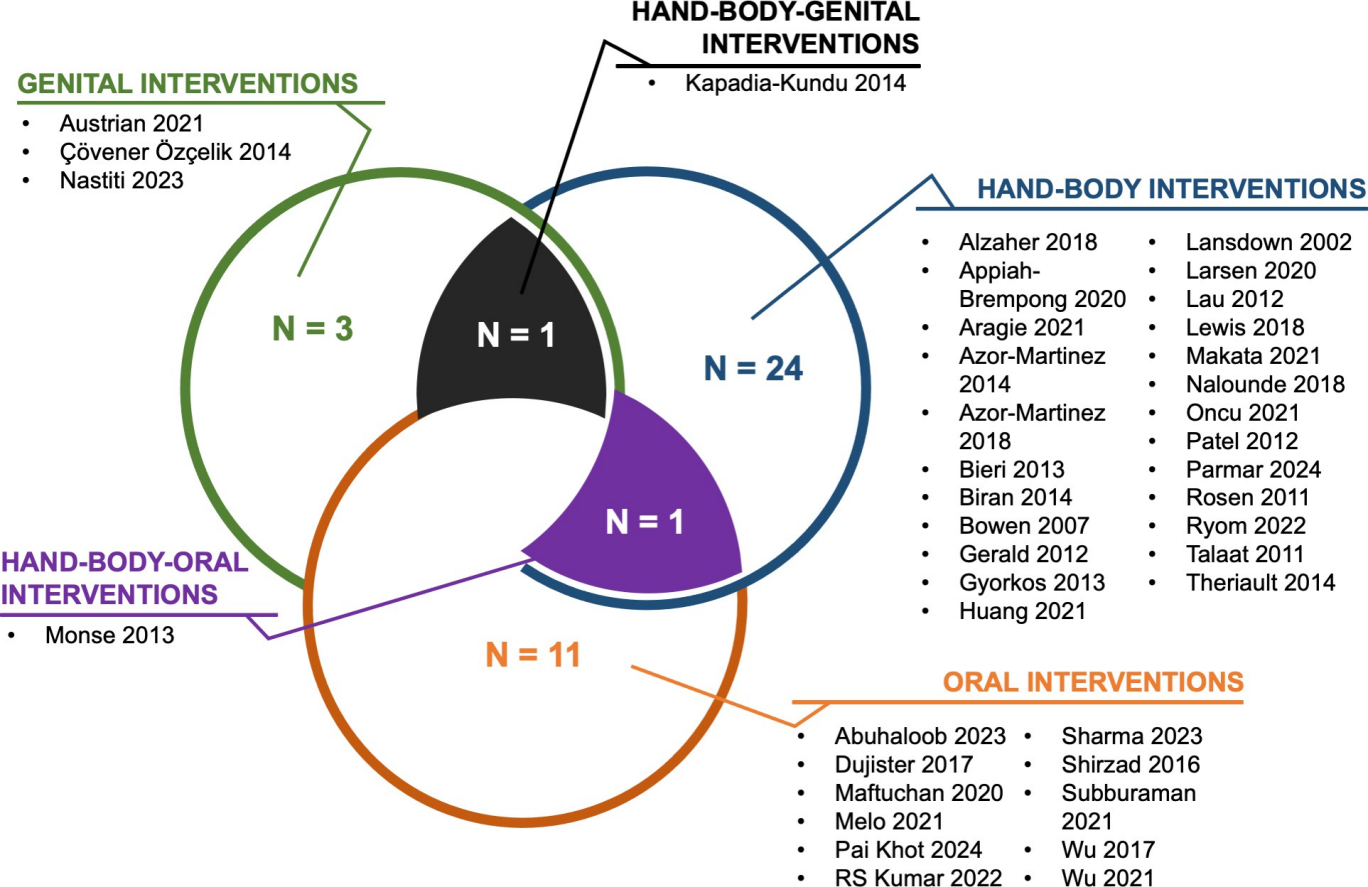
Summary of included studies by intervention. N is the number of represented studies.

We included 41 reports from 39 intervention studies (Fig 1). Larsen 2020 and Ryom 2022 were from the same intervention trial [28, 29]. Wu 2017 and Wu 2021 were also from the same trial [30, 31]. Among the included trials, 25 evaluated hand hygiene interventions, four evaluated genital hygiene interventions, and 12 evaluated oral hygiene interventions (S1–S3 Tables respectively) (Fig 2). Only one study evaluated outcomes on antimicrobial resistance [32].

### Study characteristics

Four out of 39 trials were conducted in a single school [33–36], while others included multiple schools. The smallest study was conducted in 60 children with autism in Belagavi, Karnataka, India to evaluate oral hygiene practices using a Picture Assisted Illustration Reinforcement (PAIR) communication system as compared to standard curriculum [37]. Whilst the largest included study was conducted in 60 primary schools (n = 44,451 students) in Cairo, Egypt [38]. Five studies were conducted in pre-schools, 25 in primary schools, and 12 in secondary schools or colleges.

### Intervention components

All trials (n = 39) evaluated intervention programs that included behavioural and/or educational components, and eight studies evaluated concurrently the effects of structural changes to the school facilities [8, 39–45]. Behavioural interventions included tasks that were performed repeatedly, exercises, games, puzzles, and other forms of reinforcements. Educational interventions included information-sharing sessions, talks, and lectures. Structural interventions included the construction of washing facilities such as a handwashing water station near latrines for handwashing and near classrooms for drinking and the installation of liquid soap and hand sanitiser dispensers. We summarised the interventions conducted by each study in S1 Table for hand hygiene interventions, in S2 Table for genital hygiene interventions, and in S3 Table for oral hygiene interventions.

### Geographical representation of included studies

**Fig 3:**
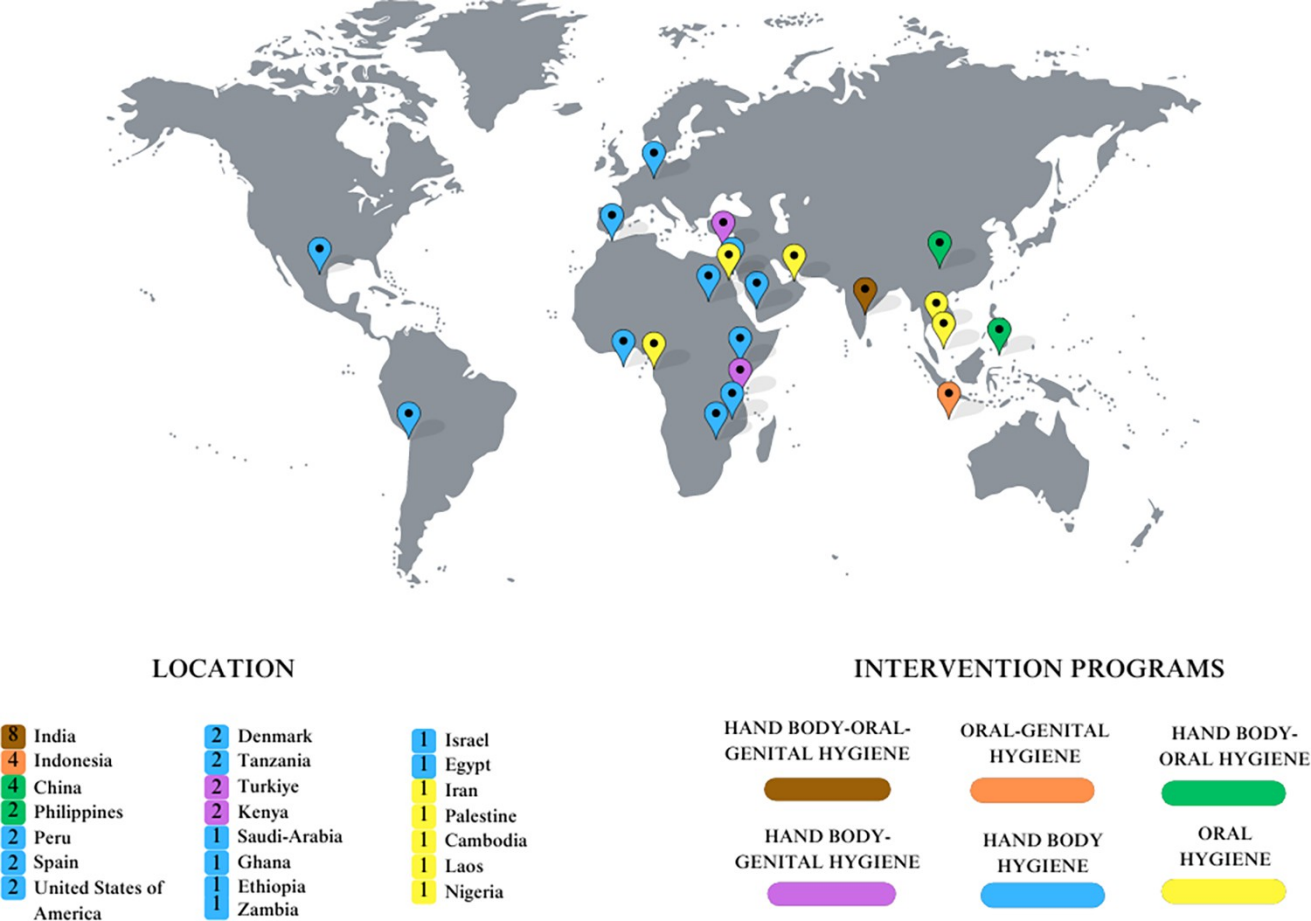
Geographical representation of included studies. Each location is represented by the types of interventions and total number of studies conducted.

Fig 3 summarises the locations of the included studies. The studies were conducted in 22 countries, including low- and middle-income countries such as India (n = 8), Indonesia (n = 4), China (n = 4), Philippines (n = 2) and Kenya (n = 2), and in developed countries such as the United States of America (n = 2), Spain (n = 2), and Denmark (n = 2).

### Risk-of-bias assessment

Among 34 cluster-RCTs, 10 were judged to have an overall high risk-of-bias, and 24 were judged as having some concerns with regards to the results of the main outcomes. There were concerns in most studies in the following domains that they might lead to bias in the results: randomisation process, deviation from the intended interventions and measurement of the outcome.

Among 6 individual RCTs, 4 were judged to have an overall high risk-of-bias and 2 as having low risk-of-bias. The main domains of concern included deviation from the intended interventions and measurement of the outcome. The single cross-over trial was judged to have some concerns overall, with bias arising from the cross-over effects and deviation from the intended interventions being the domains of concern (S3 Appendix).

### Effect estimates

#### Knowledge, attitudes and practices

**Fig 4:**
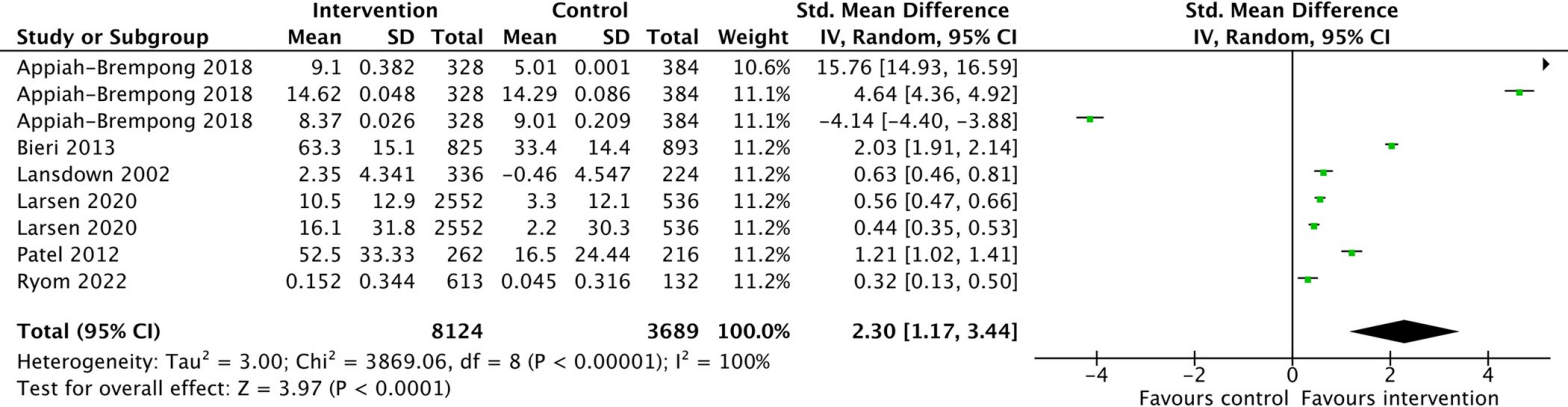
Overall knowledge, attitudes, and practices of hand hygiene interventions.

Students who participated in the hygiene intervention programs may have improved scores in overall knowledge, attitudes and practices (KAP) as compared to the control groups that underwent standard curriculum, although the evidence is presented with great uncertainty (SMD 2.30, 95%CI 1.17 to 3.44, 6 studies, 7301 participants, very-low-certainty-evidence) (Fig 4). All other forest plots of comparisons are found in S2 Appendix while the GRADE summary of findings is in Table 1.

**Table 1:** GRADE summary of hand hygiene interventions compared to standard curriculum in hand hygiene for school students.

Outcomes	№ of participants (studies) Follow-up	Certainty of the evidence (GRADE)	Relative effect (95% CI)	Anticipated absolute effects	
				Risk with SC	Risk difference with hand hygiene interventions
**Knowledge, attitudes and practices of hand hygiene**	7301 (6 RCTs)	⨁◯◯◯ Very low^1^^,^^2^^,3^	-	-	SMD **2.3 SD higher** (1.17 higher to 3.44 higher)
Assessed with knowledge, attitudes and practices surveys					
Follow-up: range 2 weeks to 2 years					
**Handwashing practices**	5479 (5 RCTs)	⨁⨁◯◯ Low^2^	**RR 1.75** (1.41 to 2.17)	673 per 1,000	**505 more per 1,000** (276 more to 788 more)
Assessed with number of handwashing events before eating and after toilet					
Follow-up: range 2 weeks to 1 years					
**School absenteeism due to infections**	1017852* (5 RCTs)	⨁⨁◯◯ Low^2^	**RR 0.74** (0.66 to 0.83)	20 per 1,000	**5 fewer per 1,000** (7 fewer to 3 fewer)
Assessed with self-reported or medically documented school absenteeism due to any infection					
Follow-up: range 5 weeks to 8 months					

The risk in the intervention group (and its 95% confidence interval) is based on the assumed risk in the comparison group and the relative effect of the intervention (and its 95% CI).

RR–risk ratio; SC–standard curriculum; SMD–standardised mean difference.

*Presented as total number of observations instead of number of participants.

^1^ Downgraded one level due to high risk of bias (Measurement outcome domain)

^2^ Downgraded two levels due to very serious concerns in inconsistency.

Given the considerable heterogeneity among studies and conflicting direction of effects observed in Fig 4, we reassessed the intervention effects to the overall KAP score, specifically focusing on interventions with durations exceeding one month. The subgroup analysis revealed smaller improvements in overall KAP scores in the intervention group participants than in the control group (SMD 0.87, 95%CI 0.31 to 1.42, 5 studies; Analysis 1.2, S2 Appendix). Appiah-Brempong 2020 has the shortest overall knowledge, attitudes and practices intervention duration of two weeks [46] as compared to other studies with longer duration of 11 weeks [28, 29] to 2 years [44].

#### Handwashing

**Fig 5:**
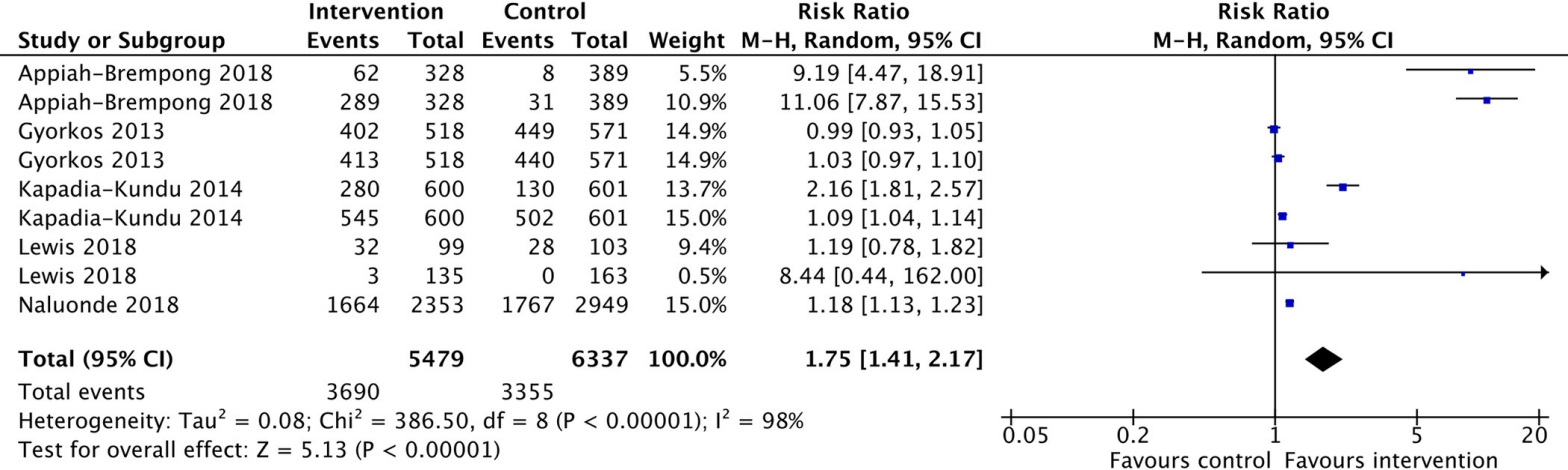
Handwashing practices.

Handwashing practices during two crucial timings were documented in the included studies: after toilet and before meals. Students in the intervention groups may be more likely to perform handwashes (with or without soap) after toilet and before meals than students in the control group (RR1.75, 95%CI 1.41 to 2.17, 5 studies, 5479 participants; low-certainty- evidence (Fig 5).

#### School absenteeism due to infections

**Fig 6:**
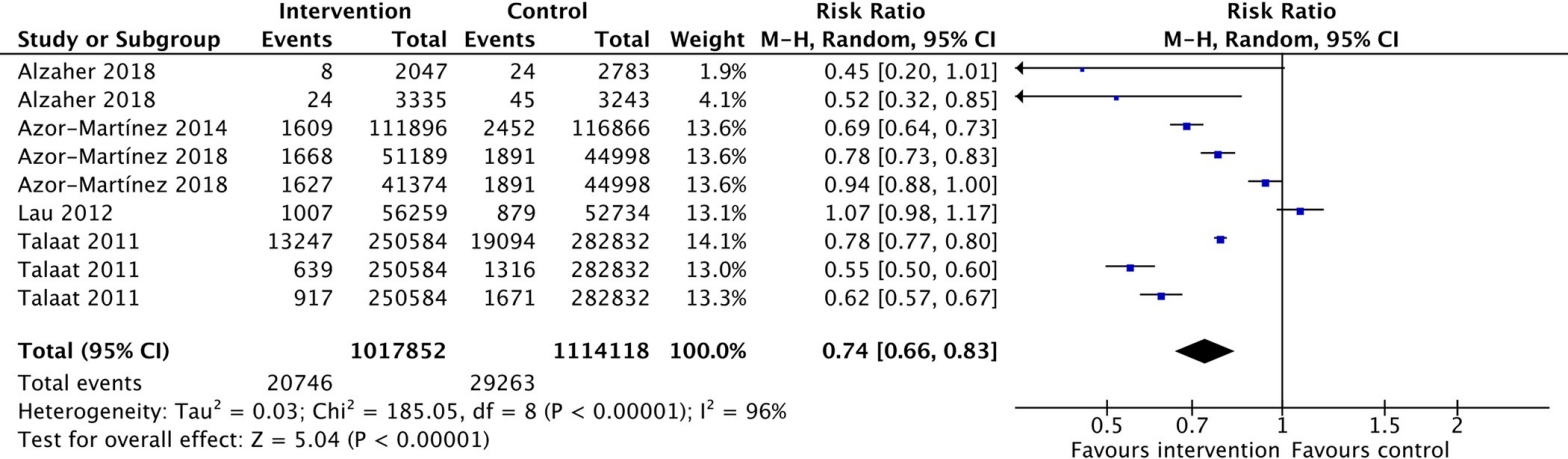
School absenteeism due to infections.

Students who underwent hand-body hygiene interventions may have a lower risk of absenteeism attributable to infections as compared to the standard curriculum (RR 0.74, 95%CI 0.66 to 0.83, 5 studies, 1017852 observations; low-certainty-evidence (Fig 6).

### Genital hygiene interventions compared to standard curriculum

Four studies reported genital hygiene interventions: Kenya (n = 1 study) [47], India (n = 1 study) [48], Indonesia (n = 1) [49] and Turkiye (n = 1 study) [33]. Three of the studies focused on menstrual hygiene in girls. The characteristics of the included studies are summarised in S2 Table.

With great uncertainty, there may be an improvement in the overall attitudes toward genital hygiene in the intervention groups as compared to the standard curriculum (SMD 6.53, 95%CI 2.40 to 10.66, 2 studies, 2644 participants, very-low-certainty-evidence) (Table 2) (Analysis 2.1 in S2 Appendix).

**Table 2:** GRADE summary of genital hygiene interventions compared to standard curriculum in genital hygiene for school students.

Outcomes	№ of participants (studies) Follow-up	Certainty of the evidence (GRADE)	Relative effect (95% CI)	Anticipated absolute effects	
				Risk with SC	Risk difference with genital hygiene interventions
**Attitude changes in genital hygiene**	2644 (2 RCTs)	⨁◯◯◯ Very low^1^^,^^2^^,^^3^	-	-	SMD **6.53 SD higher** (2.4 higher to 10.66 higher)
Assessed with genital hygiene attitude surveys					
Follow-up: range 1 month to 17 months					
**Genital hygiene practices**	1201 (1 RCT)	⨁⨁◯◯ Low^1^^,^^3^	**RR 2.44** (1.28 to 4.68)	191 per 1,000	**276 more per 1,000** (54 more to 704 more)
Assessed with genital hygiene practices surveys					
Follow-up: 1 year					

The risk in the intervention group (and its 95% confidence interval) is based on the assumed risk in the comparison group and the relative effect of the intervention (and its 95% CI).

RR–risk ratio; SC–standard curriculum; SMD–standardised mean difference.

^1^ Downgraded one level due to high risk of bias (Measurement outcome domain)

^2^ Downgraded two levels due to very serious concerns in inconsistency.

^3^ Downgraded one level due to serious concerns in imprecision.

There may also be an improvement in genital hygiene practices in students in the interventions group as compared to the students who received standard curriculum (RR 2.44, 95%CI 1.28 to 4.68, 1 study, 1201 participants, low-certainty-evidence) (Table 2) (Analysis 2.2 in S2 Appendix).

### Oral hygiene interventions compared to standard curriculum

There were ten trials (11 reports) reporting oral hygiene interventions compared to standard curriculum. Countries in which the trials were conducted include Cambodia (n = 1 study) [40], Indonesia (n = 3 studies) [40, 50, 51], India (n = 3) [37, 52, 53], Palestine (n = 1) [54], Lao PDR (n = 1 study) [40], Nigeria (n = 1 study) [51], Philippines (n = 1 study) [55], Iran (n = 1 study) [56], and China (n = 2) [30, 57].

Overall, four standardised scoring systems were used to assess oral health conditions: 1) Oral Hygiene Index Simplified (OHIS) for overall oral hygiene status; 2) Modified Gingival Index (MGI) for gum health status; 3) International Caries Detection and Assessment System (ICDAS) for dental caries; and 4) plaque score. ICDAS II 1–6 MFS and ICDAS II MFT were reported for the number of surfaces and number of teeth with dental caries (both nonactivated and cavitated lesions) [30, 57]. ICDAS II 3–6 MFS and ICDAS II 3–6 MFT were reported for the number of surfaces and number of teeth with obvious dental caries (at cavitated level) [30, 57].

#### Oral hygiene status

Oral hygiene status was assessed in four reports from three trials [30, 36, 37, 57] using at least one of the following instruments: OHIS score, Plaque score and MGI score (Table 3). Findings of one study [37], could not be quantitatively synthesised with other studies due to lack of reporting.

**Table 3:** GRADE summary of oral hygiene interventions compared to standard curriculum in oral hygiene for school students.

Outcomes	№ of participants (studies) Follow-up	Certainty of the evidence (GRADE)	Relative effect (95% CI)	Anticipated absolute effects	
				Risk with SC	Risk difference with oral hygiene interventions
**Oral hygiene status**	652 (2 RCTs)	⨁◯◯◯ Very low^1^^,^^2^	-	-	SMD **3.12 SD higher** (1.87 higher to 4.37 higher)
Assessed with OHIS score, Plaque score, MGI score					
Follow-up: range 6 months to 24 months					
**Dental hygiene status**	4824 (3 RCTs)	⨁⨁◯◯ Low^1^	-	-	SMD **0.33 SD lower** (0.53 lower to 0.13 lower)
Assessed DFMS score and DFMT score					
Follow-up: range 1 year to 2 years					
**Dental caries status**	2352 (4 RCTs)	⨁⨁◯◯ Low^1^	-	-	SMD **0.34 SD lower** (0.52 lower to 0.16 lower)
Assessed with PUFA, ICDAS II 1–6 MFT, ICDAS II 3–6 MFT, ICDAS II 1–6 MFS, ICDAS II 3–6 MFS					
Follow-up: range 1 year to 2 years					

The risk in the intervention group (and its 95% confidence interval) is based on the assumed risk in the comparison group and the relative effect of the intervention (and its 95% CI).

RR–risk ratio; SC–standard curriculum; SMD–standardised mean difference.

*Presented as total number of observations instead of number of participants.

^1^ Downgraded two levels due to very serious concerns in inconsistency.

^2^ Downgraded one level due to serious concerns in imprecision.

**Fig 7:**
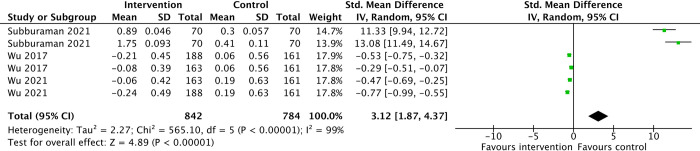
Oral hygiene status.

With interventions lasting between 3 months and 6 months, there may be an overall worsening of oral hygiene, as indicated in an increase in oral hygiene scores in the intervention groups (SMD 3.12, 95%CI 1.87 to 4.37, 652 participants, low-certainty- evidence) (Fig 7) (Table 3).

Among the three studies included, one exhibited effect in the opposite direction. To explore the factors influencing this discrepancy, we examined distinctive characteristics of the included studies and found that the age group of the study population may have been a contributing factor to these divergent effects. In analysis 3.2 of S2 Appendix, only interventions conducted in secondary school students revealed improvements in dental hygiene indexes (SMD -0.27, 95%CI -0.39 to -0.15, 2 studies) (p = 0.001 for subgroup differences).

#### Dental hygiene status

Three studies from two trials reported comparisons between oral hygiene interventions and standard curriculum. These were conducted in Palestine [54], a combined study consisting of 3 countries i.e. Cambodia, Indonesia and Lao PDR (34) and another study done in the Philippines (52).

**Fig 8:**
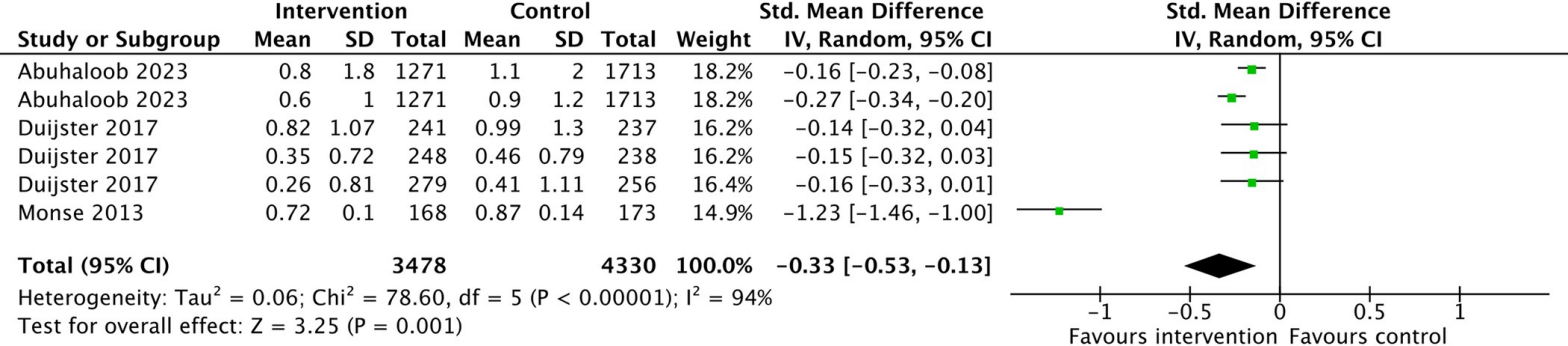
Dental hygiene status.

In comparing, oral health interventions with standard curriculum, changes in dental hygiene were reported using: 1) Decayed, Missing, and Filled Teeth/Surfaces (DFMT/DFMS) index for dental caries. There may be a modest reduction in dental caries in the intervention group (SMD -0.33, 95%CI -0.53 to -0.13, 3 studies, 4824 participants, low-certainty-evidence) (Table 3)(Fig 8).

#### Dental caries status

Three studies assessed dental caries score changes using: 1) Pulpal involvement, Ulceration, Fistula, and Abscess (PUFA) index for clinical complications of dental caries, 2) ICDAS II 1–6 MFT, 3) ICDAS II 3–6 MFT, 4) ICDAS II 1–6 MFS, and 5) ICDAS II 3–6 MFS.

These interventions were conducted in China [30, 57] while another was conducted in Cambodia, Indonesia and Lao PDR [40].

**Fig 9:**
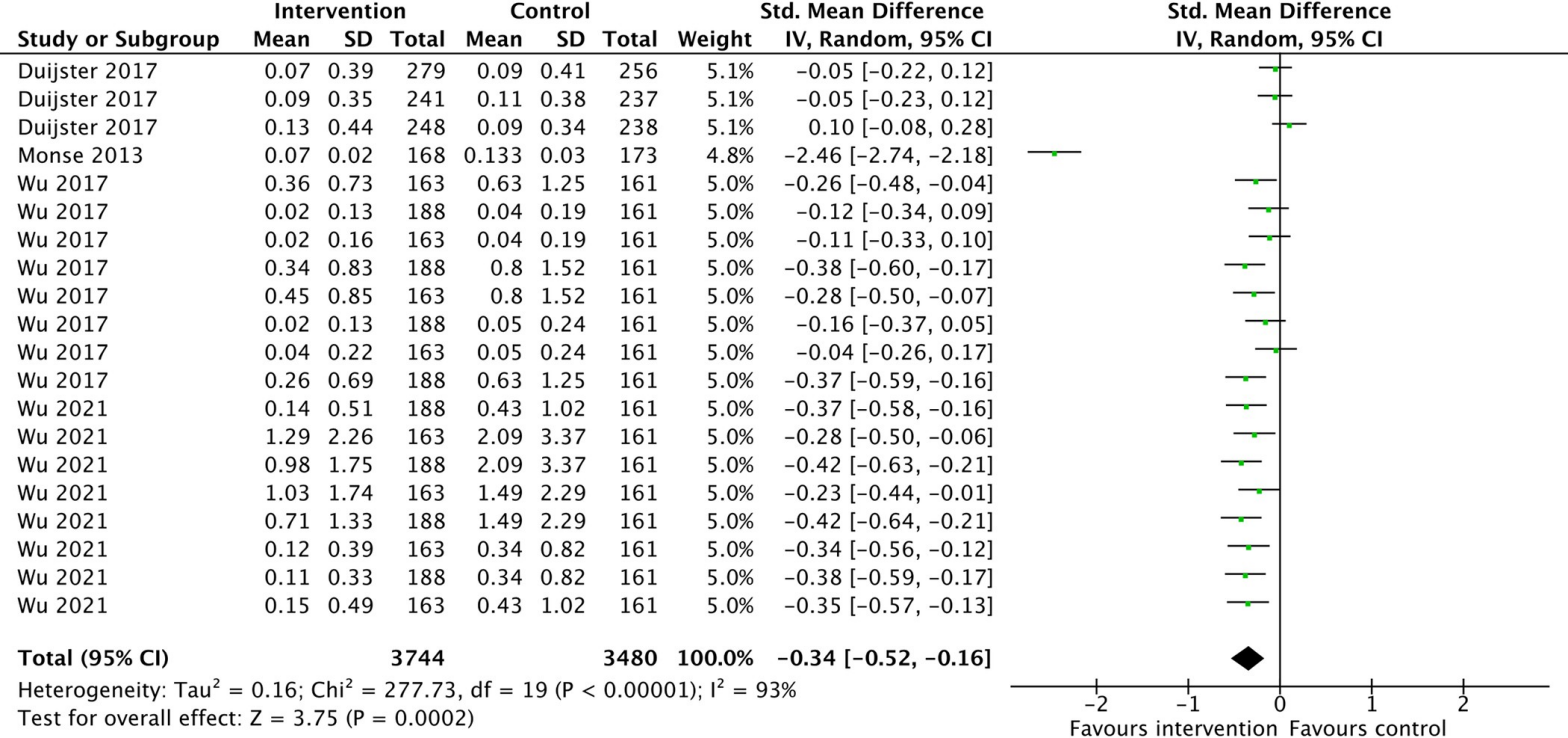
Dental caries status.

Reduction in dental caries was observed to be greater in the intervention groups than in the standard curriculum groups: (MD -0.34, 95%CI -0.52 to -0.16, 4 studies, 2352 participants, low-certainty-evidence) (Fig 9).

### Awareness of antimicrobial resistance

There is one study that evaluated antimicrobial resistance, which did not provide suitable outcome data for meta-analysis. The study was a non-randomised intervention study in high school students which compared a 15-min lecture on antimicrobial resistance and antibiotic used using United States Centres for Disease and Prevention and Control and the World Health Organisation materials, with standard curriculum [32].

The intervention group demonstrated significant improvements of participants’ knowledge of antibiotics and causes of antibiotics resistance before and after intervention as compared to the control group (p<0.001). There were also significant improvements of participants’ perceptions towards antibiotic resistance in the intervention group as compared to the control group (p<0.001).

## Discussion

### Summary of the main results

In this review, we found very low-to-low-certainty evidence that school-based interventions may improve overall knowledge, attitude and practice on general, hand and genital hygiene and reduce school absenteeism. The evidence on oral and dental hygiene status was mixed when measured using different tools. However, in most estimates, the effects varied among studies as indicated by high heterogeneity, and this was inadequately explained. Besides heterogeneity, the evidence is affected by the risk-of-bias of the studies and imprecision, as some outcomes were contributed by the small number of studies. These factors have led to a reduction in the certainty of the evidence presented.

### Agreement and disagreement with similar reviews

At least eight published reviews have evaluated school-based hygiene-related interventions. However, as far as we are aware, none of the published reviews covered the interventions in the same scope as the current review. Two reviews, each of 8 studies, that evaluated hand hygiene promotion interventions in schools reported potential modest benefits, but with low-quality evidence due mainly to study methodological limitations and conflicting results among included studies [58, 59]. Two reviews, including a Cochrane review that evaluated the use of rinse-free sanitizers in schools reported reduced school absenteeism in the intervention group but low-quality of evidence due to methodological shortcomings of the studies included [60, 61]. A review of 3 studies found suboptimal adherence to hand hygiene in schools (<50%), with educators demonstrating higher adherence than students [62]. School-based oral health interventions in India (10 articles) consistently reported improvements in oral hygiene among school children following the interventions. However, the evidence was limited due to methodological concerns in the included studies [63]. Evidence on Water, Sanitation and Hygiene (WASH) initiatives in schools was mixed, with some studies showing improvements in knowledge, attitude and behaviours, but no consistent significant differences in health outcomes like diarrhoea [23].

A broad Cochrane review on the WHO Health Promoting School framework covering healthy lifestyles found insufficient evidence on the effects of intervention on hand washing practice and oral health, with studies having low-to-moderate quality evidence and high risk of bias [64]. Compared to the aforementioned reviews that evaluated a specific intervention, for example, hand hygiene or oral health promotion, the current review is broader in scope and encompasses all hygiene-related school-based interventions. Compared with other reviews that evaluated comprehensive healthy lifestyle intervention, our review is narrower in scope, focussing only on hygiene related interventions. However, the findings of our review are broadly in agreement with the aforementioned reviews, in showing modest benefit of low-to-very-low certainty for some of the hygiene related interventions and mixed findings for other interventions, highlighting methodological limitations of the included studies.

### Strengths and limitations of the review

We performed comprehensive searches from multiple databases and gathered a globally representative sample of studies conducted from 2011 to 2023 across 22 countries in America, Europe, Africa and Asia, making the evidence widely applicable. The systematic review was conducted using established methodologies, including the most updated version of the risk-of-bias tool and certainty-of-evidence assessments.

We acknowledge the following limitations. The main issue of concern in the review is the marked heterogeneity in the pooled estimates. Possible sources for the high degree of heterogeneity may be the specific details in population characteristics, intervention design, in particular, specific components of the intervention and comparator, including settings, methods, duration of administration and specific timing of assessments. More in-depth assessments of these factors are required, including among others, adherence to interventions and the reliability of self-reported outcome measures. However, these aspects were often not clearly defined and documented in the included studies. Next, the current review might not have captured a complete set of relevant studies. Despite a careful assessment of a broad set of nearly 500 studies that appeared relevant, we might have missed eligible studies, for instance, studies that evaluated interventions predominantly implemented outside the school setting or not clearly labelled as school-based but contained important components in the school. Next, in dealing with multiple reports, we performed careful assessments and merged the reports based on the reported study period, participant characteristics and settings. Despite our careful assessment, there remains a possibility of inappropriate combination of studies. Next, we deviated from our protocol in the following aspects: first, in the protocol, we included schoolteachers as our participants. In the review, we decided to focus on the students and exclude schoolteachers, as we did not find any paper that evaluated teachers’ perception or knowledge following our current search strategies. To include studies that evaluated only teachers would probably have required a revision of our search strategies. Next, we decided to focus on the studies that evaluated the effects of hygiene intervention, rather than including non-interventional studies that evaluated knowledge, attitude, and practice, as well as the prevalence of hygiene-related practice. Although the protocol deviation might have led mainly to a narrowing of the scope of our evidence, there is a possibility that by excluding pre-specified participants and study types, we might have excluded relevant evidence.

Additionally, we acknowledge that the considerable variability in study design, implementation and evaluation of the school-based hygiene interventions impact the applicability of the study findings. Firstly, the interventions range from comprehensive multidimensional interactive sessions to structural changes to the school such as installing handwashing stations. The variability reflects the different contexts and needs of the study populations but would also limit its interpretation and comparison of findings across studies. Secondly, the duration and intensity of the interventions varied widely across the studies. Duration is an important predictor of behavioural interventions. Longer interventions generally provide more time for the students to learn, practice, and reinforce new behaviours, which is a crucial for habit formation and skill development. To reduce these effects, we performed several sub-group analyses based on duration of interventions. This allowed for a more comparable outcome across different studies. Thirdly, the methods used to evaluate knowledge, attitude and practices (KAP) differed across the studies. Although this raises concerns about the ability to draw definitive conclusions from varying tools, it is important to note that KAP questionnaires should be tailored to the study population. This ensures relevance to the age, maturity, cultural, social, and environmental contexts of the study populations. Tailored questionnaires provide more meaningful and accurate data, which enhances the validity and reliability of the findings.

While we do recognise the importance of clear and thorough documentation and reporting practices in research, we believe that the large majority of the included studies demonstrated detailed methodologies, particularly in the assessment of outcomes. Therefore, poor documentation and reporting practices as a critical issue in this study. Nonetheless, we acknowledge that any instances of poor reporting and documentation of the included studies could impact the reliability of the outcomes and thus necessitate interpretation with caution.

While this study focuses on school-based interventions, we acknowledge the valuable insights provided by community-based interventions in child and adolescent health. Community-based programs also play a significant role in promoting healthy behaviours and can complement school-based efforts. However, the structured environment of schools offers unique opportunities for consistent and supportive interactions that are essential for habit formation and long-term behaviour change. Therefore, our emphasis on school-based interventions highlights their critical role in the holistic development of children.

### Addressing methodological limitations in future research

We had previously acknowledge that methodological limitations of the included studies contribute to the overall uncertainty of our findings. The variability in intervention design and implementation, including differences in duration and delivery methods, poses challenges for drawing definitive conclusions and comparing outcomes across studies. Additionally, many studies had a high or unclear risk of bias in key domains, such as the randomisation process and measurement of outcomes, which can affect the reliability of the results. The varying measurement tools across the studies also contributed to the heterogeneity of the findings. This limits the comprehensive assessment of the interventions.

Based on the limitations previously described, future studies should be aimed at providing a more robust evidence for the effectiveness of school-based hygiene interventions. This includes standardisation of intervention protocols, standardisation of measurement tools for different outcomes and specific for different age groups, and longer follow-up periods to assess the sustained impact of interventions. Addressing these issues will enhance the quality and comparability of the findings.

## Conclusion

Very-low-to-low-certainty-evidence shows that among school children, educational interventions targeting hand hygiene practice may improve knowledge and reduce school absenteeism due to respiratory and diarrheal illness. Genital hygiene educational intervention may improve knowledge and attitude based on mostly low-certainty-evidence. However, the effects of intervention on oral hygiene-related outcomes appeared mixed. Future research should strengthen randomisation and intervention documentation, and evaluate the association between knowledge gain, hygiene-related behaviour and health outcomes including school absenteeism, school academic performances and severity of infections.

## Supporting information

S1 Table Characteristics of studies with hand-body hygiene intervention programs. (DOCX)

S2 Table Characteristics of studies with genital hygiene intervention programs. (DOCX)

S3 Table Characteristics of studies with oral hygiene intervention programs. (DOCX)

S1 Appendix Search strategy. (DOCX)

S2 Appendix Summary of all meta-analysis. (DOCX)

S3 Appendix Summary of risk of bias assessment of included studies. (DOCX)

## References

[1] CDC. Personal Hygiene: Centers for disease control and prevention (CDC) 2023 [cited 2023 4 November 2023]. Available from: https://www.cdc.gov/hygiene/personal-hygiene/index.html.

[2] Vivas AP, Gelaye B, Aboset N, Kumie A, Berhane Y, Williams MA. Knowledge, attitudes and practices (KAP) of hygiene among school children in Angolela, Ethiopia. Journal of preventive medicine and hygiene. 2010;51(2):73–9. 21155409 PMC3075961

[3] Cha YE, Fu YZ, Yao W. Knowledge, Practice of Personal Hygiene, School Sanitation, and Risk Factors of Contracting Diarrhea among Rural Students from Five Western Provinces in China. International journal of environmental research and public health. 2021;18(18). 34574432 10.3390/ijerph18189505 PMC8468795

[4] Gbogbo S, Amu H, Dowou RK, Ayanore MA. Management of onchocerciasis among adolescents in nkwanta north district of Ghana: Qualitative study of adolescents’ perception, community and health system support. PLoS Negl Trop Dis. 2023;17(8):e0011577. doi: 10.1371/journal.pntd.0011577 37643153 PMC10464985

[5] Lopez-Quintero C, Freeman P, Neumark Y. Hand washing among school children in Bogotá, Colombia. American journal of public health. 2009;99(1):94–101. 19008513 10.2105/AJPH.2007.129759 PMC2636611

[6] UNICEF. Childhood diseases: UNICEF; 2023 [cited 2023 4 November 2023]. Available from: https://www.unicef.org/health/childhood-diseases.

[7] Azor-Martinez E, Cobos-Carrascosa E, Seijas-Vazquez ML, Fernández-Sánchez C, Strizzi JM, Torres-Alegre P, et al. Hand Hygiene Program Decreases School Absenteeism Due to Upper Respiratory Infections. The Journal of school health. 2016;86(12):873–81. doi: 10.1111/josh.12454 27866386

[8] Azor-Martínez E, Gonzalez-Jimenez Y, Seijas-Vazquez ML, Cobos-Carrascosa E, Santisteban-Martínez J, Martínez-López JM, et al. The impact of common infections on school absenteeism during an academic year. American journal of infection control. 2014;42(6):632‐7. doi: 10.1016/j.ajic.2014.02.017 24837113

[9] Sandora TJ, Shih MC, Goldmann DA. Reducing absenteeism from gastrointestinal and respiratory illness in elementary school students: a randomized, controlled trial of an infection-control intervention. Pediatrics. 2008;121(6):e1555–62. doi: 10.1542/peds.2007-2597 18519460

[10] Tsang TK, Huang X, Guo Y, Lau EHY, Cowling BJ, Ip DKM. Monitoring School Absenteeism for Influenza-Like Illness Surveillance: Systematic Review and Meta-analysis. JMIR public health and surveillance. 2023;9:e41329. doi: 10.2196/41329 36630159 PMC9878370

[11] Pinkerton R, Oriá RB, Lima AA, Rogawski ET, Oriá MO, Patrick PD, et al. Early Childhood Diarrhea Predicts Cognitive Delays in Later Childhood Independently of Malnutrition. The American journal of tropical medicine and hygiene. 2016;95(5):1004–10. doi: 10.4269/ajtmh.16-0150 27601523 PMC5094207

[12] Maillard JY, Bloomfield SF, Courvalin P, Essack SY, Gandra S, Gerba CP, et al. Reducing antibiotic prescribing and addressing the global problem of antibiotic resistance by targeted hygiene in the home and everyday life settings: A position paper. Am J Infect Control. 2020;48(9):1090–9. doi: 10.1016/j.ajic.2020.04.011 32311380 PMC7165117

[13] Adebisi YA, Alaran AJ, Okereke M, Oke GI, Amos OA, Olaoye OC, et al. COVID-19 and Antimicrobial Resistance: A Review. Infectious diseases. 2021;14:11786337211033870. doi: 10.1177/11786337211033870 34376994 PMC8327234

[14] Getahun H, Smith I, Trivedi K, Paulin S, Balkhy HH. Tackling antimicrobial resistance in the COVID-19 pandemic. Bulletin of the World Health Organization. 2020;98(7):442-a. doi: 10.2471/BLT.20.268573 32742026 PMC7375214

[15] Lynch C, Mahida N, Gray J. Antimicrobial stewardship: a COVID casualty? The Journal of hospital infection. 2020;106(3):401–3. doi: 10.1016/j.jhin.2020.10.002 33045312 PMC7545211

[16] WHO. Coronavirus disease (COVID-19) pandemic: World Health Organisation (WHO) 2023 [cited 2023 2 November 2023]. Available from: https://www.who.int/emergencies/diseases/novel-coronavirus-2019.

[17] UNICEF W. Progress on drinking water, sanitation and hygiene in schools: 2000–2021 Data update: WHO/UNICEF Joint Monitoring Programme (JMP) for Water Supply, Sanitation and Hygiene (WASH); 2021 [cited 2023 2 November 2023]. The WHO/UNICEF Joint Monitoring Programme (JMP) produces internationally comparable estimates of progress on drinking water, sanitation and hygiene (WASH) and is responsible for monitoring the Sustainable Development Goal (SDG) targets related to WASH. This data update presents national, regional and global estimates for WASH in schools up to the year 2021, and includes additional analysis on efforts to prepare schools for future pandemics and provide disability-inclusive WASH services in schools.]. Available from: https://data.unicef.org/resources/jmp-wash-in-schools-2022/.

[18] UNICEF. Water, Sanitation and Hygiene (WASH) Implementation for Schools and Healthcare facilities: United Nations Department of Economic and Social Affairs 2022 [cited 2023 2 November 2023]. Available from: https://sdgs.un.org/partnerships/water-sanitation-and-hygiene-wash-implementation-schools-and-healthcare-facilities#targets-tab.

[19] Magiorakos AP, Suetens C, Boyd L, Costa C, Cunney R, Drouvot V, et al. National Hand Hygiene Campaigns in Europe, 2000–2009. Eurosurveillance. 2009;14(17):19190. 19422767

[20] CDC. Infection Control: Centers for Disease Control and Prevention (CDC); 2023 [cited 2023 4 November 2023]. Available from: https://www.cdc.gov/infectioncontrol/index.html.

[21] WHO. Global strategy on infection prevention and control: World Health Organisation (WHO) 2023 [cited 2023 4 November 2023]. Available from: https://www.who.int/publications/m/item/global-strategy-on-infection-prevention-and-control.

[22] UKHSA. e-Bug: UK Health Security Agency 2023 [cited 2023 4 November 2023]. Available from: https://www.e-bug.eu/.

[23] McMichael C. Water, Sanitation and Hygiene (WASH) in Schools in Low-Income Countries: A Review of Evidence of Impact. International journal of environmental research and public health. 2019;16(3). doi: 10.3390/ijerph16030359 30696023 PMC6388361

[24] WHO U. > Core questions and indicators for monitoring WASH in Schools in the Sustainable Development Goals: JMP UNICEF and World Health Organization; 2018 [cited 2023 2 November 2023]. This document presents recommended core questions to support harmonised monitoring of WASH in schools as part of the SDGs. The questions map to harmonised indicator definitions of “basic” service and to service ladders that can be used to monitor progress. They are intended for use in national or sub-national facility surveys and census questionnaires. If national and sub-national surveys use the questions and response categories in this guide, it will help to improve survey comparability over time and between countries, as well as harmonise data with the SDG definitions for WASH in schools.]. Available from: https://www.washinschoolsindex.com/document/148.

[25] Jasper C, Le TT, Bartram J. Water and sanitation in schools: a systematic review of the health and educational outcomes. International journal of environmental research and public health. 2012;9(8):2772–87. doi: 10.3390/ijerph9082772 23066396 PMC3447586

[26] Sangalang SO, Lemence ALG, Ottong ZJ, Valencia JC, Olaguera M, Canja RJF, et al. School water, sanitation, and hygiene (WaSH) intervention to improve malnutrition, dehydration, health literacy, and handwashing: a cluster-randomised controlled trial in Metro Manila, Philippines. BMC Public Health. 2022;22(1):2034. doi: 10.1186/s12889-022-14398-w 36344973 PMC9641834

[27] Sterne JAC, Savović J, Page MJ, Elbers RG, Blencowe NS, Boutron I, et al. RoB 2: a revised tool for assessing risk of bias in randomised trials. BMJ. 2019;366:l4898. doi: 10.1136/bmj.l4898 31462531

[28] Larsen MN, Elbe AM, Madsen M, Madsen EE, Ørntoft C, Ryom K, et al. An 11-week school-based ’health education through football programme’ improves health knowledge related to hygiene, nutrition, physical activity and well-being-and it’s fun! A scaled-up, cluster-RCT with over 3000 Danish school children aged 10–12 years old. Br J Sports Med. 2021;55(16):906–11. doi: 10.1136/bjsports-2020-103097 33509911

[29] Ryom K, Christiansen SR, Elbe AM, Aggestrup CS, Madsen EE, Madsen M, et al. The Danish "11 for Health" program raises health knowledge, well-being, and fitness in ethnic minority 10- to 12-year-olds. Scand J Med Sci Sports. 2022;32(1):138–51. doi: 10.1111/sms.14057 34555200

[30] Wu L, Gao X, Lo ECM, Ho SMY, McGrath C, Wong MCM. Motivational Interviewing to Promote Oral Health in Adolescents. Journal of Adolescent Health. 2017;61(3):378–84. doi: 10.1016/j.jadohealth.2017.03.010 28532895

[31] Wu S, Wang RS, Huang YN, Wan TTH, Tung TH, Wang BL. Effect of Hand Hygiene Intervention in Community Kindergartens: A Quasi-Experimental Study. International journal of environmental research and public health. 2022;19(22). doi: 10.3390/ijerph192214639 36429356 PMC9691028

[32] Almutairi KS, Okmi EA, Alnofaiei SS, Alshamari WK, Almutairi SH, Alsuwailem SI, et al. The Effects of Health Education on the Awareness of Antimicrobial Resistance Among High School Students in Riyadh, Saudi Arabia During 2023: A Quasi-experimental Study. Cureus. 2023;15(7):e41639. doi: 10.7759/cureus.41639 37565089 PMC10411380

[33] Cövener Özçelik C, Aktaş E, Celik D, Ocakçı AF. Impact of toilet hygiene training program: results from 11- to 16-year-old secondary school Turkish children. International journal of public health. 2014;59(5):799–807. 25173963 10.1007/s00038-014-0595-1

[34] Kumar RS, Deshpande AP, Ankola AV, Sankeshwari RM, Jalihal S, Hampiholi V, et al. Effectiveness of a Visual Interactive Game on Oral Hygiene Knowledge, Practices, and Clinical Parameters among Adolescents: A Randomized Controlled Trial. Children (Basel). 2022;9(12). 10.3390/children9121828 PMC9776468 36553272

[35] Öncü E, Vayısoğlu SK. Duration or technique to improve the effectiveness of children’ hand hygiene: A randomized controlled trial. Am J Infect Control. 2021;49(11):1395–401. doi: 10.1016/j.ajic.2021.03.012 33774105

[36] Subburaman N, Parangimalai DM, Iyer K, Sukumaran A. Effectiveness of social media based oral health promotion programme among 18–20 year old city college students—A comparative study. Indian J Dent Res. 2021;32(4):467–71. doi: 10.4103/ijdr.ijdr_1128_20 35645073

[37] Pai Khot AJ, Choudhury AR, Ankola AV, Sankeshwari RM, Hampiholi V, Hebbal M, et al. Evaluation of a “Picture Assisted Illustration Reinforcement” (PAIR) System for Oral Hygiene in Children with Autism: a Double-Blind Randomized Controlled Trial. Children. 2023;10(2). doi: 10.3390/children10020369 36832499 PMC9955023

[38] Talaat M, Afifi S, Dueger E, El-Ashry N, Marfin A, Kandeel A, et al. Effects of hand hygiene campaigns on incidence of laboratory-confirmed influenza and absenteeism in schoolchildren, Cairo, Egypt. Emerging infectious diseases. 2011;17(4):619–25. doi: 10.3201/eid1704.101353 21470450 PMC3377412

[39] Azor-Martinez E, Yui-Hifume R, Muñoz-Vico FJ, Jimenez-Noguera E, Strizzi JM, Martinez-Martinez I, et al. Effectiveness of a Hand Hygiene Program at Child Care Centers: A Cluster Randomized Trial. Pediatrics. 2018;142(5). 10.1542/peds.2018-1245 30297500

[40] Duijster D, Monse B, Dimaisip-Nabuab J, Djuharnoko P, Heinrich-Weltzien R, Hobdell M, et al. ’Fit for school’—a school-based water, sanitation and hygiene programme to improve child health: results from a longitudinal study in Cambodia, Indonesia and Lao PDR. BMC public health. 2017;17(1):302. doi: 10.1186/s12889-017-4203-1 28381246 PMC5382467

[41] Gerald LB, Gerald JK, Zhang B, McClure LA, Bailey WC, Harrington KF. Can a school-based hand hygiene program reduce asthma exacerbations among elementary school children? The Journal of allergy and clinical immunology. 2012;130(6):1317–24. doi: 10.1016/j.jaci.2012.08.031 23069487 PMC3511646

[42] Lau CH, Springston EE, Sohn M-W, Mason I, Gadola E, Damitz M, et al. Hand hygiene instruction decreases illness-related absenteeism in elementary schools: a prospective cohort study. BMC Pediatrics. 2012;12(1):52. doi: 10.1186/1471-2431-12-52 22587432 PMC3470997

[43] Makata K, Ensink J, Ayieko P, Hansen C, Sichalwe S, Mngara J, et al. Hand hygiene intervention to optimise soil-transmitted helminth infection control among primary school children: the Mikono Safi cluster randomised controlled trial in northwestern Tanzania. BMC medicine. 2021;19(1):125. doi: 10.1186/s12916-021-01987-6 34016091 PMC8139108

[44] Patel MK, Harris JR, Juliao P, Nygren B, Were V, Kola S, et al. Impact of a hygiene curriculum and the installation of simple handwashing and drinking water stations in rural Kenyan primary schools on student health and hygiene practices. The American journal of tropical medicine and hygiene. 2012;87(4):594–601. doi: 10.4269/ajtmh.2012.11-0494 22869631 PMC3516306

[45] Rosen L, Zucker D, Brody D, Engelhard D, Meir M, Manor O. Enabling hygienic behavior among preschoolers: improving environmental conditions through a multifaceted intervention. American journal of health promotion. 2011;25(4):248‐56. doi: 10.4278/ajhp.081104-QUAN-265 21361810

[46] Appiah-Brempong E, Newton S, Harris MJ, Gulis G. Effect of a theory-based hand hygiene educational intervention for enhancing behavioural outcomes in Ghanaian schools: a cluster-randomised controlled trial. International journal of public health. 2020;65(1):99–109. doi: 10.1007/s00038-019-01310-4 31701186

[47] Austrian K, Kangwana B, Muthengi E, Soler-Hampejsek E. Effects of sanitary pad distribution and reproductive health education on upper primary school attendance and reproductive health knowledge and attitudes in Kenya: a cluster randomized controlled trial. Reproductive health [Internet]. 2021; 18(1):[179 p.]. Available from: https://www.cochranelibrary.com/central/doi/ doi: 10.1186/s12978-021-01223-7 34465344 PMC8406733

[48] Kapadia-Kundu N, Storey D, Safi B, Trivedi G, Tupe R, Narayana G. Seeds of prevention: the impact on health behaviors of young adolescent girls in Uttar Pradesh, India, a cluster randomized control trial. Social science & medicine (1982). 2014;120:169‐79. doi: 10.1016/j.socscimed.2014.09.002 25254614

[49] Nastiti AA, Triharini M, Pratiwi AH, Kamel Gouda AD. Educational intervention to improve menstrual hygiene management in adolescent girls in Kalimantan, Indonesia. J Pak Med Assoc. 2023;73(Suppl 2)(2):S13–s7. doi: 10.47391/JPMA.Ind-S2-3 37096693

[50] Maftuchan M, Hadi EN. Kiddie Doctors Education Strategy in Improving the Knowledge, Attitude, and Oral Hygiene Status of Elementary School Children. Journal of International Society of Preventive & Community Dentistry. 2020;10(5):549–54. doi: 10.4103/jispcd.JISPCD_116_20 33282762 PMC7685274

[51] Melo P, Fine C, Malone S, Taylor S. Impact of the Brush Day & Night Programme on Well-Being, Plaque, and Dental Caries in Children. International dental journal [Internet]. 2021. Available from: https://www.cochranelibrary.com/central/doi/10.1002/central/CN-02260977/full. 10.1016/j.identj.2021.01.018 PMC9275125 33678399

[52] Ram Surath Kumar K, Despande AP, Ankola AV, Sankeshwari RM, Jalihal S, Hampiholi V, et al. Effectiveness of a Visual Interactive Game on Oral Hygiene Knowledge, Practices, and Clinical Parameters among Adolescents: A Randomized Controlled Trial. Children (Basel, Switzerland). 2022;9(12). 10.3390/children9121828 PMC9776468 36553272

[53] Sharma M, Bachani R. Knowledge, Attitude, Practice, and Perceived Barriers for the Compliance of Standard Precautions among Medical and Nursing Students in Central India. International journal of environmental research and public health. 2023;20(8). doi: 10.3390/ijerph20085487 37107771 PMC10139079

[54] Abuhaloob L, Petersen PE. Health-Promoting Schools Project for Palestine Children’s Oral Health. Int Dent J. 2023. doi: 10.1016/j.identj.2023.03.011 37120392 PMC10509424

[55] Monse B, Benzian H, Naliponguit E, Belizario V, Schratz A, van Palenstein Helderman W. The Fit for School Health Outcome Study—a longitudinal survey to assess health impacts of an integrated school health programme in the Philippines. BMC public health. 2013;13:256. doi: 10.1186/1471-2458-13-256 23517517 PMC3610233

[56] Shirzad M, Taghdisi MH, Dehdari T, Abolghasemi J. Oral health education program among pre-school children: an application of health-promoting schools approach. Health promotion perspectives. 2016;6(3):164–70. doi: 10.15171/hpp.2016.26 27579261 PMC5002884

[57] Wu L, Lo ECM, McGrath C, Wong MCM, Ho SMY, Gao X. Motivational interviewing for caries prevention in adolescents: a randomized controlled trial. Clinical Oral Investigations. 2022;26(1):585–94. doi: 10.1007/s00784-021-04037-w 34254214

[58] Watson JA, Ensink JHJ, Ramos M, Benelli P, Holdsworth E, Dreibelbis R, et al. Does targeting children with hygiene promotion messages work? The effect of handwashing promotion targeted at children, on diarrhoea, soil-transmitted helminth infections and behaviour change, in low- and middle-income countries. Tropical medicine & international health: TM & IH. 2017;22(5):526–38. 10.1111/tmi.12861 28244191

[59] Mbakaya BC, Lee PH, Lee RL. Hand Hygiene Intervention Strategies to Reduce Diarrhoea and Respiratory Infections among Schoolchildren in Developing Countries: A Systematic Review. International journal of environmental research and public health. 2017;14(4). doi: 10.3390/ijerph14040371 28368323 PMC5409572

[60] Meadows E, Le Saux N. A systematic review of the effectiveness of antimicrobial rinse-free hand sanitizers for prevention of illness-related absenteeism in elementary school children. BMC Public Health. 2004;4:50. doi: 10.1186/1471-2458-4-50 15518593 PMC534108

[61] Munn Z, Tufanaru C, Lockwood C, Stern C, McAneney H, Barker TH. Rinse-free hand wash for reducing absenteeism among preschool and school children. The Cochrane database of systematic reviews. 2020;4(4):Cd012566. doi: 10.1002/14651858.CD012566.pub2 32270476 PMC7141998

[62] Resende KKM, Neves LF, de Rezende Costa Nagib L, Martins LJO, Costa CRR. Educator and Student Hand Hygiene Adherence in Dental Schools: A Systematic Review and Meta-Analysis. Journal of dental education. 2019;83(5):575–84. 30804172 10.21815/JDE.019.060

[63] Gambhir RS, Sohi RK, Nanda T, Sawhney GS, Setia S. Impact of school based oral health education programmes in India: a systematic review. Journal of clinical and diagnostic research: JCDR. 2013;7(12):3107–10. 24551745 10.7860/JCDR/2013/6212.3718 PMC3919396

[64] Langford R, Bonell CP, Jones HE, Pouliou T, Murphy SM, Waters E, et al. The WHO Health Promoting School framework for improving the health and well‐being of students and their academic achievement. Cochrane Database of Systematic Reviews. 2014(4). doi: 10.1002/14651858.CD008958.pub2 24737131 PMC11214127

