# Supplementary material for: The effects of school-based hygiene intervention programme: Systematic review and meta-analysis
Source: PLoS One. 2024 Oct 8;19(10):e0308390. doi: 10.1371/journal.pone.0308390 (PMC11460677; doi:10.1371/journal.pone.0308390)
Supplement: S1 Table — (DOCX) [file pone.0308390.s001.docx]

**HYGIENE SYSTEMATIC REVIEW**

**S1 Table: Characteristics of studies with hand-body hygiene intervention programs**

| **Study, Country** | **Total Randomised** | **Participants** | **Components of the intervention program** | **Control** | **Duration of participation** |
| --- | --- | --- | --- | --- | --- |
| Alzaher 2018, Saudi Arabia | 4 schools  496 students | Primary school girls aged 6 - 12 years old | 1. 6-minute video-clip 2. Short interactive lecture 3. Puzzle games 4. Posters | Standard curriculum | 5 weeks |
| Appiah-Brempong 2020, Ghana | 4 schools  717 students | Grades 7, 8 and 9 school students | 1. Handwashing KAP workshops 2. Posters | Standard curriculum | 2 weeks |
| Aragie 2021, Ethiopia | 40 primary-school clusters | Primary school students aged 6 - 9 years | 1. Teaching aids 2. Instruction manual for extracurricular clubs dedicated to promoting hygiene at the school and in the community. | Standard curriculum | 3 years |
| Azor-Martínez 2014, Spain | 5 schools  1,616 students | Primary school students aged 4 to 12 years old | 1. Two-hour handwashing workshop for pupils and teachers 2. Activities such as stories and songs 3. Posters 4. Brochures 5. Hand sanitizer dispensers | Standard curriculum | 8 months |
| Azor-Martinez 2018, Spain | 25 daycare clusters  1,176 children | Daycare center children | Soap-and-water (SWG) group:   1. One-hour hand hygiene workshop 2. Hand-washing procedures before/after activities throughout the day 3. Informational brochure 4. Hygiene-related activities such as stories and songs 5. Posters 6. Supply of liquid soap for use in classrooms and at home.   Hand-sanitizer (HSG) group:   1. Same hand hygiene promotional program as SWG group except for the supply of liquid soaps 2. Supply of hand sanitizers for use in classrooms and at home | A 1-hour hand hygiene workshop followed by standard curriculum | 8 months |
| Bieri 2013, China | 38 schools  1,934 children | Grade 4 and 5 school students | 1. 12-minute cartoon 2. Classroom discussions 3. Posters 4. Pamphlets 5. Competitions on soil-transmitted helminths infection prevention 6. Deworming at the beginning of the program | Deworming followed by standard curriculum | 7 months |
| Biran 2014, India | 15 villages  348 households | Primary school students aged between 8 and 13 years old | 1. Super Amma animated film 2. Skits contrasting the clean habits of Super Amma with her dirty comic counterpart. 3. Interim activities. | Shorter duration of the same activities | Intervention group = 25 days  Control group = 9 days |
| Bowen 2007, China | 87 schools  3,810 students | First-grade school students | Standard Intervention:   1. Standard government hygiene educational programming 2. Classroom teaching sessions 3. Animated videotape 4. Student's take-home pack (hygiene board game, parents' booklet about handwashing, and a 50g bar of Safeguard bar soap) 5. Teacher's pack (guidebook, five handwashing posters, and five classroom hygiene competition wall charts).   Expanded Intervention Group:   1. Same handwashing promotion program as standard intervention group 2. Continuous supply of Safeguard bar soap for use at school sinks 3. Recruitment of one student from each first-grade class to assist peers with handwashing techniques and to remind them of key handwashing opportunities while at school | Standard curriculum | 10 weeks |
| Gerald 2012, USA | 31 schools  527 students | Elementary school students | Two-step hand hygiene intervention followed by standard curriculum.  Two-step hand hygiene intervention:   1. Supply and installation of alcohol-based hand sanitizer, hand soap, and refills 2. Installation of hand soap dispensers in the schools’ restrooms 3. Hand hygiene education | Standard curriculum is followed by the same two-step hand hygiene intervention. | 10 months for each intervention with a crossover during the summer period |
| Gyorkos 2013, Peru | 18 schools  1,486 children | Grade 5 primary school students | 1. Deworming at the beginning of the program 2. One-hour classroom health education activities guided by a customized booklet. 3. Half-day workshop for teachers and school principals 4. Educational posters. | Regular deworming program | 4 months |
| Huang 2021, Philippines | 132 schools | Primary school students | 1. Contextual cues (such as painted footpath/arrow stickers) 2. Visual reminders (such as posters, and eye stickers) | Standard curriculum | 4 months |
| Kapadia-Kundu 2014, India | 30 schools  1,200 girls | Grade 6,7 and 8 schoolgirls | The Saloni pilot intervention:   1. Saloni Diary – activities, areas to track hygiene and nutrition changes, and document aspirations and long-term goals 2. Promotion of daily handwashing with soap 3. Promotion of daily genital hygiene 4. Promotion of changing home-made sanitary napkins thrice daily during menstruation 5. Promotion of intergenerational communication with household members 6. Standard curriculum (Saloni adolescent health program) | Standard curriculum (Saloni adolescent health program):   1. Weekly supply of iron and folic acid tables 2. Annual health checkups 3. Six-monthly deworming doses 4. Counselling session | 1 year |
| Lansdown 2002, Tanzania | 50 schools  560 students | Primary school students aged between 7 and 15 years old | 1. Two workshops for schoolteachers on active teaching methods of health education. 2. Each school produced materials that had been developed by children and teachers, including songs, stories, and pictures | Standard curriculum | 5 months |
| Larsen 2020, Denmark | 154 schools  6,132 children | Schoolchildren aged 10 - 12 years old | A locally adapted Danish version of the ‘11 for Health’ program:   1. Health education 2. Football drills 3. Small-sided games | Standard curriculum | 11 weeks |
| Lau 2012, USA | 2 schools  981 students | Kindergarten to Grade 8 students | 1. Supply of hand sanitizer and hand washing facilities at the school 2. Hand sanitizer promotional posters 3. Hand sanitizer usage protocol 4. Grade-appropriate curriculum (interactive and review sessions on hand hygiene) | 1. Supply of hand sanitizer and hand washing facilities at the school 2. Hand sanitizer promotional posters 3. 30-minute lesson on hand hygiene at the end of the study | 8 months |
| Lewis 2018, India | 32 villages  75 schools  338 households | Primary school students | Unilever's "School of 5" program:   1. Interactive activities 2. 'Enrolment cards' to each student for telling up to 10 relatives or friends what they learned during the sessions. 3. Student pledge for handwashing with soap on target occasions 4. Meeting with the mothers | Standard curriculum | 21 days |
| Makata 2021, Tanzania | 16 school  9,479 students | Primary school students | 1. Deworming at the beginning of the program 2. Handwashing with water and soap during classroom lessons 3. Demonstrations 4. Games 5. Parents engagement 6. Modest modifications to the handwashing facilities in school | Deworming followed by standard curriculum | 1 year |
| Monse 2013, Philippines | 4 schools  412 students | Grade 1 school students | Philippine Essential Health Care Program:   1. Supervised group handwashing with soap and clean water activity 2. Supervised brushing with a fluoride toothpaste group activity 3. Biannual deworming | Standard curriculum | 4 years |
| Naluonde 2018, Zambia | 50 schools  10,732 students | Primary and secondary school students | Soap-On-A-Rope (SOAR) was a handwashing cue with a small piece of soap hanging on a piece of rope or cord that serves as a hall pass | Standard curriculum followed by SOAR | Intervention group = 3 months  Control group = 1-month standard curriculum and 2 months SOAR |
| Öncü 2021, Turkiye | 155 students | Grade 4 school students | Intervention Group 1:   1. A 15-minute of classroom training on handwashing 2. Installation of handwashing banners in classrooms 3. Pictural cards of the WHO's multi-staged handwashing technique 4. Demonstration and repeated supervision of WHO 11-steps of multi-staged handwashing technique.   Intervention Group 2:   1. A 15-minute of classroom training on handwashing 2. Installation of handwashing banners in classrooms 3. Pictural cards of the CDC handwashing recommendations 4. Demonstration and supervision of CDC handwashing recommendations. | 1. A 15-minute classroom training on handwashing 2. Installation of handwashing banners in classrooms 3. Pictural cards on the importance of hand cleaning. | 14 days |
| Parmar 2024, India | 20 daycare centres,  800 children | Aged between 1 and 5 years | 1. Handwashing- incorporated structured handwashing sessions with soap and water 2. Hand sanitizers- utilized alcohol-based hand sanitizers   Educational program- Educational Program | Standard curriculum | 6 months |
| Patel 2012, Kenya | 60 villages  43 schools  643 households  783 students | Grades 4 to 8 school students | Nyando Integrated Child Health and Education (NICHE):   1. Training of teachers on handwashing and water treatment 2. Installation of water station for handwashing near latrines 3. Installation of water station for drinking near classrooms 4. A 3-month “starter” supply of soap and WaterGuard water treatment solution | No intervention in Year 1. The same NICHE intervention was provided in Year 2 | 2 years |
| Rosen 2011, Israel | 40 schools  1,029 students | Preschool students | 1. Installation of dispensers and supply of supplies for handwashing 2. Health education training sessions for teachers, assistants, and nurses 3. Educational kit with games and self-reward charts for children 4. Hand hygiene education with a puppeteer 5. Individual demonstration of proper hand washing 6. Home kit (a video of the program, a card, and a magnet) | Standard curriculum | 3 months |
| Ryom 2022, Denmark | 154 schools  1,122 students | Ethnic minority school children aged 10 - 12 years old | A locally adapted Danish version of the ‘11 for Health’ program:   1. Health education 2. Football drills 3. Small-sided games | Standard curriculum | 11 weeks |
| Talaat 2011, Egypt | 60 schools  44,451 students | Grades 1 to 3 of primary school students | 1. Regular hand washing activity in school 2. Supply of teachers’ guidebook with students’ activities 3. Posters 4. Grade-specific student booklets with handwashing games and activities 5. Hand hygiene promotional song 6. Informational fliers to parents 7. Other school-based initiatives | Standard curriculum | 12 weeks |
| Theriault 2014, Peru | 18 schools  1,486 students | Grade 5 primary school students | 1. Booklets 2. Posters 3. Interactive activities 4. Weekly lectures to the students 5. Workshops for the teachers on STH transmission and prevention 6. Deworming at the beginning of the program | Deworming followed by standard curriculum | 4 months |

Abbreviations: KAP – knowledge, attitudes, and practices; STH – soil-transmitted helminths; USA – United States of America.
