# Supplementary material for: The effects of school-based hygiene intervention programme: Systematic review and meta-analysis
Source: PLoS One. 2024 Oct 8;19(10):e0308390. doi: 10.1371/journal.pone.0308390 (PMC11460677; doi:10.1371/journal.pone.0308390)
Supplement: S2 Table — (DOCX) [file pone.0308390.s002.docx]

**HYGIENE SYSTEMATIC REVIEW**

**S2 Table: Characteristics of studies with genital hygiene intervention programs.**

| **Study, Country** | **Total Randomized** | **Participants** | **Components of the intervention program** | **Control** | **Duration of participation** |
| --- | --- | --- | --- | --- | --- |
| Austrian 2021, Kenya | 140 schools  3,489 students | Grade 7 schoolgirls | Pads only group   1. Monthly supply of sanitary pads   Reproductive health group   1. Reproductive health education and magazine   Combined group   1. Monthly supply of sanitary pads 2. Reproductive health education and magazine | Standard curriculum | 17 months |
| Çövener Özçelik 2014, Turkiye | 100 students | Secondary school male and female students | 1. Toilet hygiene education presentation | Standard curriculum | One month |
| Kapadia-Kundu 2014, India | 30 schools  1,200 girls | Grade 6,7 and 8 schoolgirls | The Saloni pilot intervention:   1. Saloni Diary – activities, areas to track hygiene and nutrition changes, and document aspirations and long-term goals 2. Promotion of daily handwashing with soap 3. Promotion of daily genital hygiene 4. Promotion of changing home-made sanitary napkins thrice daily during menstruation 5. Promotion of intergenerational communication with household members 6. Usual program (Saloni adolescent health program) | Saloni adolescent health program:   1. Weekly supply of iron and folic acid tablets 2. Annual health checkups 3. Six-monthly deworming doses 4. Counseling session | 1 year |
| Nastiti 2023, Indonesia | 70 girls | Grade 7 female students | 1. Health education intervention through a 90-minute video conferences in two meetings 2. Health education leaflet | Health education leaflet | 4 months |
