# Supplementary material for: The effects of school-based hygiene intervention programme: Systematic review and meta-analysis
Source: PLoS One. 2024 Oct 8;19(10):e0308390. doi: 10.1371/journal.pone.0308390 (PMC11460677; doi:10.1371/journal.pone.0308390)
Supplement: S3 Table — (DOCX) [file pone.0308390.s003.docx]

**HYGIENE SYSTEMATIC REVIEW**

**S3 Table: Characteristics of studies with oral hygiene intervention programs**

| **Study, Country** | **Total Randomized** | **Participants** | **Components of the intervention program** | **Control** | **Duration of participation** |
| --- | --- | --- | --- | --- | --- |
| Abulahoob 2023, Palestine | 61 schools  2,984 students | Grade 1 primary school students aged between 5 and 6 years old | 1. Provision of fluoride containing toothpaste and toothbrushes 2. Daily supervised toothbrushing at school 3. Oral health education school events at school for both parents and children 4. Regular communication between teachers and parents on children's oral health 5. Comprehensive capacity-building workshops for teachers | Standard curriculum | 2 years |
| Dujister 2017, Cambodia | 20 schools  478 students | Grade 1 primary school students aged between 6 and 7 years old | FIT program:   1. Daily group handwashing with soap activity 2. Daily group toothbrushing activity 3. Biannual deworming 4. Construction and maintenance of group washing facilities | Standard curriculum and biannual deworming | 24 months |
| Dujister 2017, Indonesia | 18 schools  486 students | Grade 1 primary school students aged between 6 and 7 years old | FIT program:   1. Daily group handwashing with soap activity 2. Daily group toothbrushing activity 3. Biannual deworming 4. Construction and maintenance of group washing facilities | Standard curriculum and biannual deworming | 24 months |
| Dujister 2017, Lao PDR | 44 schools  535 students | Grade 1 primary school students aged between 6 and 7 years old | FIT program:   1. Daily group handwashing with soap activity 2. Daily group toothbrushing activity 3. Biannual deworming 4. Construction and maintenance of group washing facilities | Standard curriculum and biannual deworming | 24 months |
| Maftuchan 2020, Indonesia | 2 schools  143 students | Grade 3, 4, and 5 school students | 1. Regular oral health education program 2. Kiddie Doctor Activity Guide Module for Elementary School Children which trained the selected students to provide oral hygiene education to their peers through information sharing, games, and demonstrations. | Standard curriculum | 4 months |
| Melo 2021, Indonesia | 22 schools  2,021 students | Grades 1,2 and 3 school students | Brush Day & Night program:   1. Provision of toothbrush and toothpaste 2. Singing and supervision during toothbrushing 3. Progress tracking with stickers and calendars followed by celebration with certificates and rewards. 4. Educational leaflets for parents | Provision of toothbrush and toothpaste at baseline followed by standard curriculum | 21 days |
| Melo 2021, Nigeria | 20 schools  1,947 students | Grades 1,2 and 3 school students | Brush Day & Night program:   1. Provision of toothbrush and toothpaste 2. Singing and supervision during toothbrushing 3. Progress tracking with stickers and calendars followed by celebration with certificates and rewards. 4. Educational leaflets for parents | Provision of toothbrush and toothpaste at baseline followed by standard curriculum | 21 days |
| Monse 2013, Philippines | 4 schools  412 students | Grade 1 school students | Philippine Essential Health Care Program:   1. Supervised group handwashing with soap and clean water activity 2. Supervised brushing with a fluoride toothpaste group activity 3. Biannual deworming | Standard curriculum | 4 years |
| Pai Khot 2023, India | 2 schools  60 participants | Children and adolescents aged 7 to 18 years old | The Picture Assisted Illustration Reinforcement (PAIR) technique   1. Illustration and identification of the pictures by the children. 2. Children were handed the illustrated object in exchange for choosing the 3. correct picture. 4. Children identify both the correct and incorrect illustrations that were displayed. 5. Children frame a sentence following the sequence of the pictures. | Standard curriculum   1. Oral health education talk to children and school teachers 2. Demonstration of Fones method of toothbrushing | 6 months |
| Ram Surath Kumar 2022, India | 100 students | Primary school students aged between 12 and 15 years old | 1. A customized animated video which included introduction to oral health and its significance in general health, the brushing technique, and the five golden rules for maintaining effective oral health 2. A 30 min online game-based question and answer interactive session. 3. Brushing technique (Modified Bass Technique) training | A 15min conventional oral health education (oral health introduction and five golden rules for maintaining effective oral health)  Brushing technique (Modified Bass Technique) demonstration | One-off |
| Sharma 2023, India | 3 schools  120 students | Students aged between 12 and 14 years old with Autism Spectrum Disorder (ASD). | Dental health education by trained teachers (1hour weekly):   1. Dental health education (oral diseases – causes, progression, treatment and prevention) 2. Brushing technique demonstration 3. Health education aids - brushing models, pamphlets and charts   Dental health education by trained students (1hour weekly):   1. Dental health education (oral diseases – causes, progression, treatment and prevention) 2. Brushing technique demonstration 3. Health education aids - brushing models, pamphlets and charts | Dental health education by trained dentist once:   1. Dental health education (oral diseases – causes, progression, treatment and prevention) 2. Brushing technique demonstration 3. Health education aids - brushing models, pamphlets and charts | 3 months |
| Shirzad 2016, Iran | 4 schools  120 students | Preschool girls aged 5 - 6 years old | 1. Separate interactive oral health education sessions for the students and parents/teachers 2. Educational booklets for parents and teachers | Standard curriculum | 6 months |
| Subburaman 2021, India | 140 students | College students aged 18 - 20 years | 1. Oral health education lecture, peer teaching, and demonstrations using tooth models at the beginning of the study. 2. Reinforcement of oral health education through WhatsApp covering different monthly themes. | Oral health education lecture, peer teaching, and demonstrations using tooth models at the beginning of the study. | 3 months |
| Wu 2017, China | 15 schools  512 students | Secondary school students aged either 12 or 13 years old | Group 1:   1. Face-to-face motivational interviewing at the beginning 2. Scheduled follow-up telephone calls   Group 2:   1. Face-to-face motivational interviewing at the beginning 2. Scheduled follow-up telephone calls 3. Interactive dental caries risk assessment program at different stages. | 1. Health talk 2. Discussion with dental hygienist 3. Pamphlets | 6 months |
| Wu 2021, China | 15 schools  512 participants | Secondary school students aged either 12 or 13 years old | Group 1:   1. Face-to-face motivational interviewing at the beginning 2. Scheduled follow-up telephone calls   Group 2:   1. Face-to-face motivational interviewing at the beginning 2. Scheduled follow-up telephone calls 3. Interactive dental caries risk assessment program at different stages. | 1. Health talk 2. Discussion with dental hygienist 3. Pamphlets | 6 months |
