# Supplementary material for: The effects of school-based hygiene intervention programme: Systematic review and meta-analysis
Source: PLoS One. 2024 Oct 8;19(10):e0308390. doi: 10.1371/journal.pone.0308390 (PMC11460677; doi:10.1371/journal.pone.0308390)
Supplement: S1 Appendix — (DOCX) [file pone.0308390.s004.docx]

**HYGIENE SYSTEMATIC REVIEWS**

**S1 Appendix Search Strategy**

The following search strategy was applied for MEDLINE (PubMed). We adapted the same search strategy for CENTRAL.

#1 antibiotic*[Title/Abstract]

#2 antimicrobial[Title/Abstract]

#3 "antimicrobial stewardship"[MeSH Terms]

#4 hygiene[MeSH Terms]

#5 "hygiene/education"[MeSH Terms]

#6 hygien*[Title/Abstract]

#7 #1 OR #2 OR #3 OR #4 OR #5 OR #6

#8 “pre-school” [Title/Abstract]

#9 "primary school"[Title/Abstract]

#10 "secondary school"[Title/Abstract]

#11 "high school"[Title/Abstract]

#12 college*[Title/Abstract]

#13 school-age[Title/Abstract]

#14 "school children"[Title/Abstract]

#15 "school health services/education"[MeSH Terms]

#16 "educational personnel"[MeSH Terms]

#17 school teacher[MeSH Terms]

#18 "student health services"[MeSH Terms]

#19 "students"[MeSH Terms]

#20 teacher*[Title/Abstract]

#21 #8 OR #9 OR #10 OR #11 OR #12 OR #13 OR #14 OR #15 OR #16 OR #17 OR #18 OR #19 OR #20

#22 #7 AND #21
