# Supplementary material for: The effects of school-based hygiene intervention programme: Systematic review and meta-analysis
Source: PLoS One. 2024 Oct 8;19(10):e0308390. doi: 10.1371/journal.pone.0308390 (PMC11460677; doi:10.1371/journal.pone.0308390)

**HYGIENE SYSTEMATIC REVIEW**

**S2 Appendix Summary of all meta-analysis**

## Comparison 1: Hand hygiene interventions versus standard curriculum

### Analysis 1.1. Overall knowledge, attitudes and practices of hand hygiene


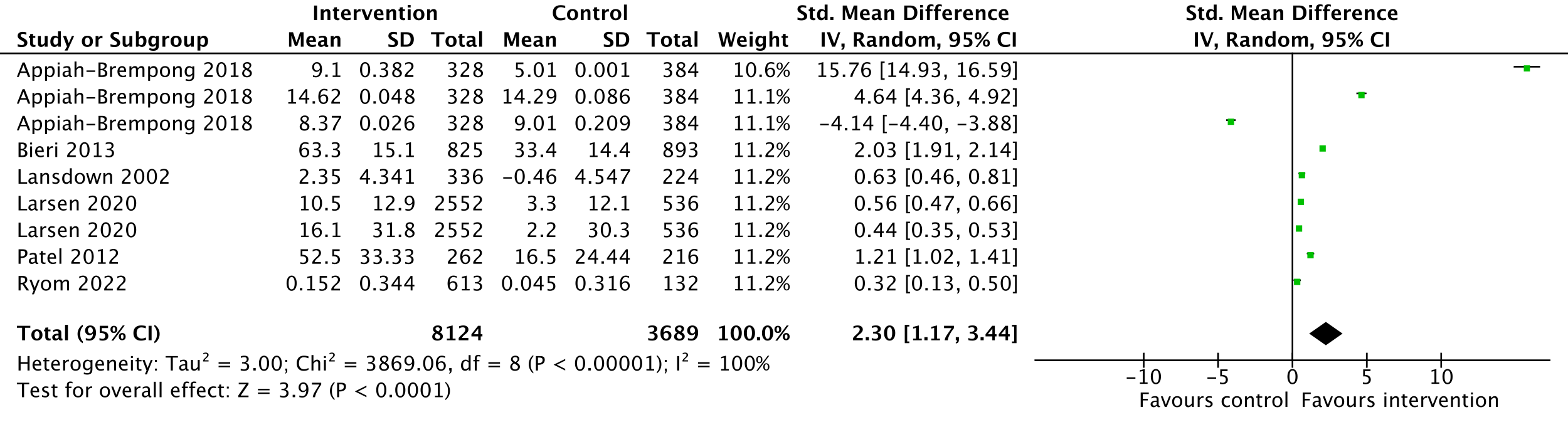


### Analysis 1.2. Subgroup analysis for interventions lasting >1 month for overall knowledge, attitudes and practices of hand hygiene.


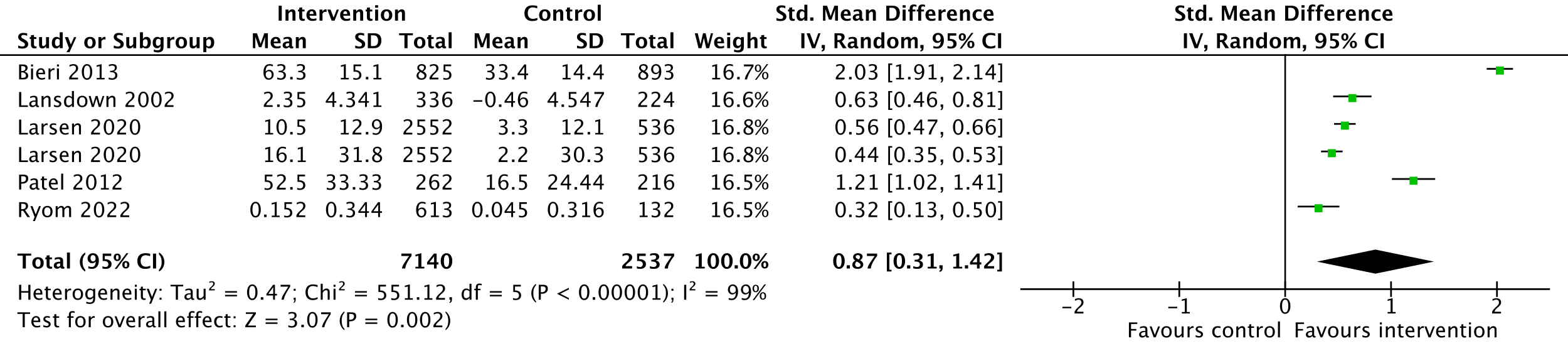


### Analysis 1.3. Knowledge of hand hygiene


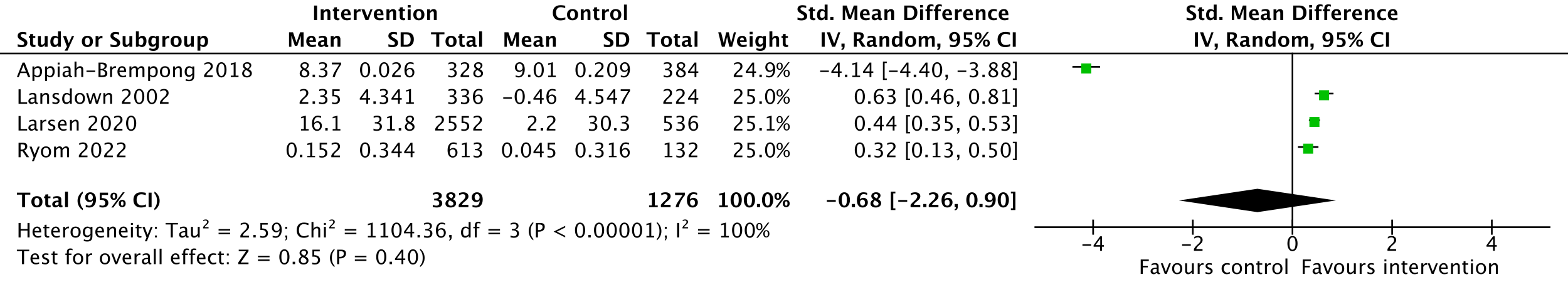


### Analysis 1.4. Subgroup analysis for interventions lasting >1 month for knowledge of hand hygiene.


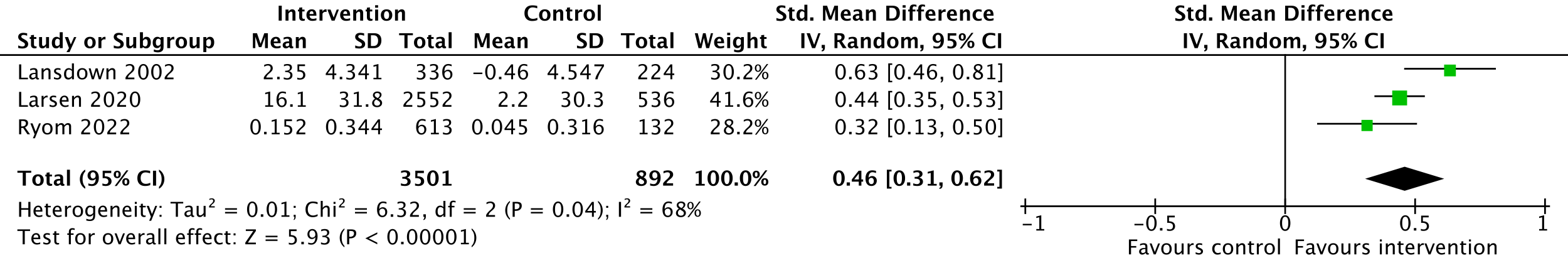


### Analysis 1.5. Practices of hand hygiene (Survey)


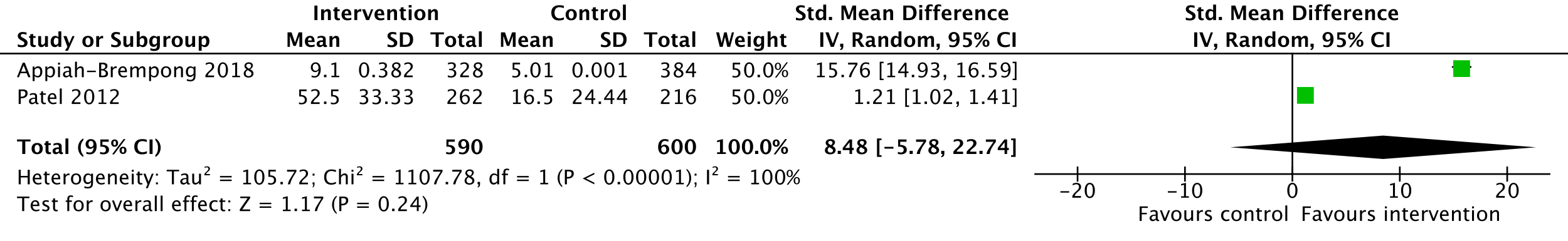


### Analysis 1.6. Overall handwashing practices


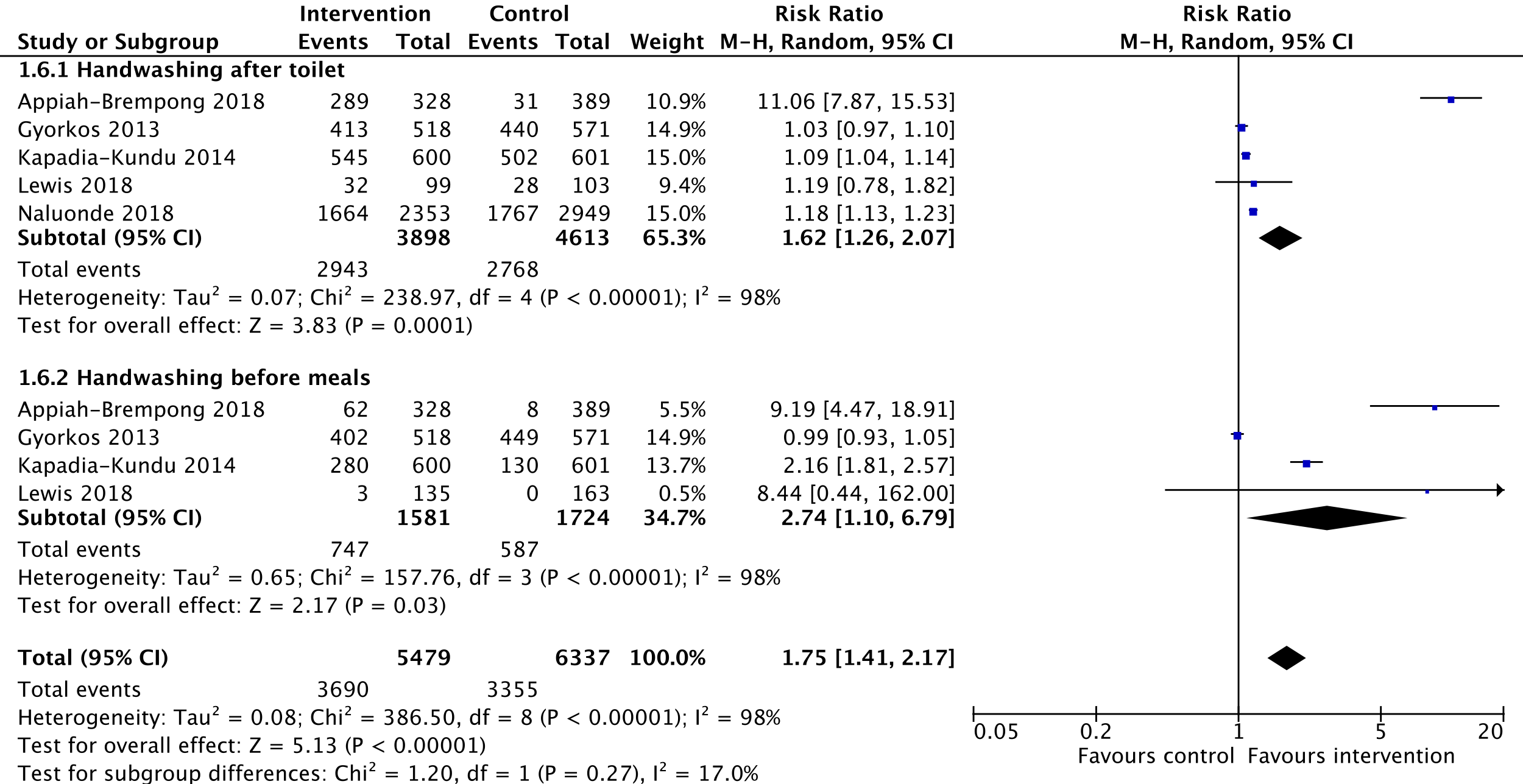


### Analysis 1.7. Subgroup analysis for handwashing practices after toilet


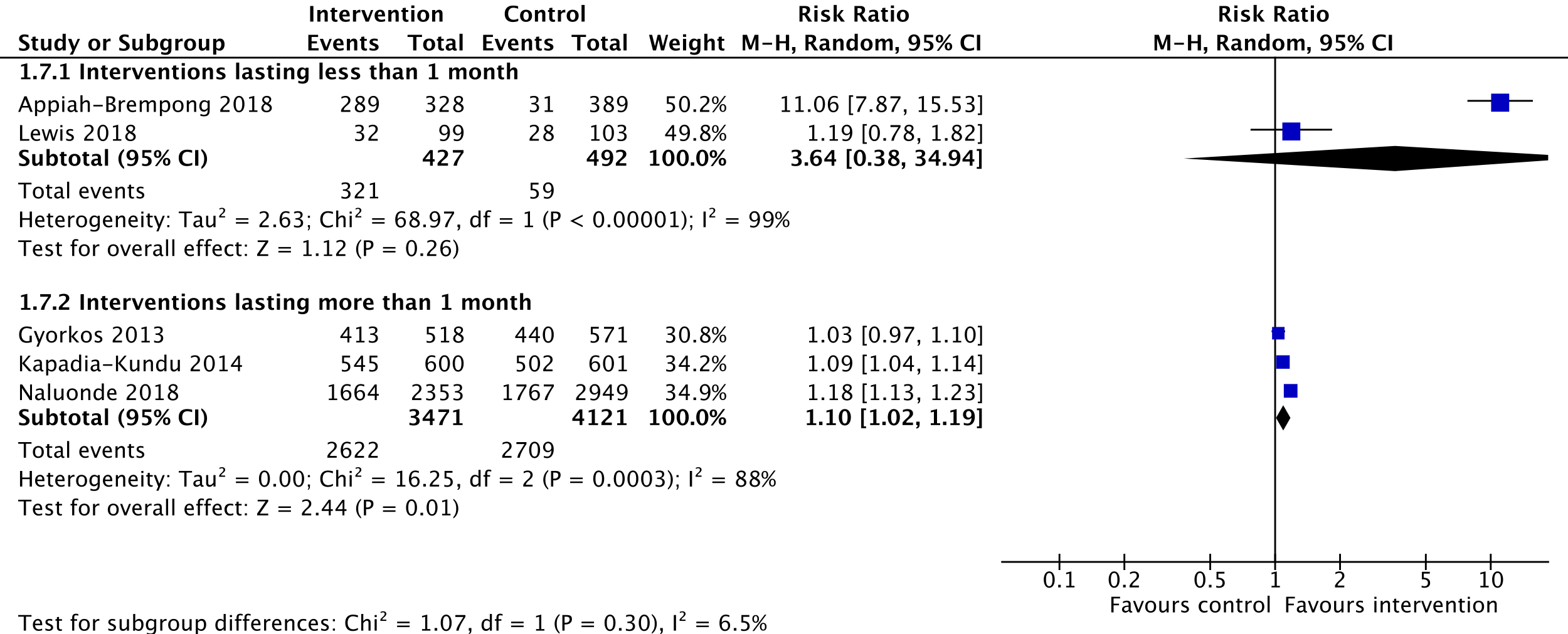


### Analysis 1.8. Subgroup analysis for handwashing practices before meals


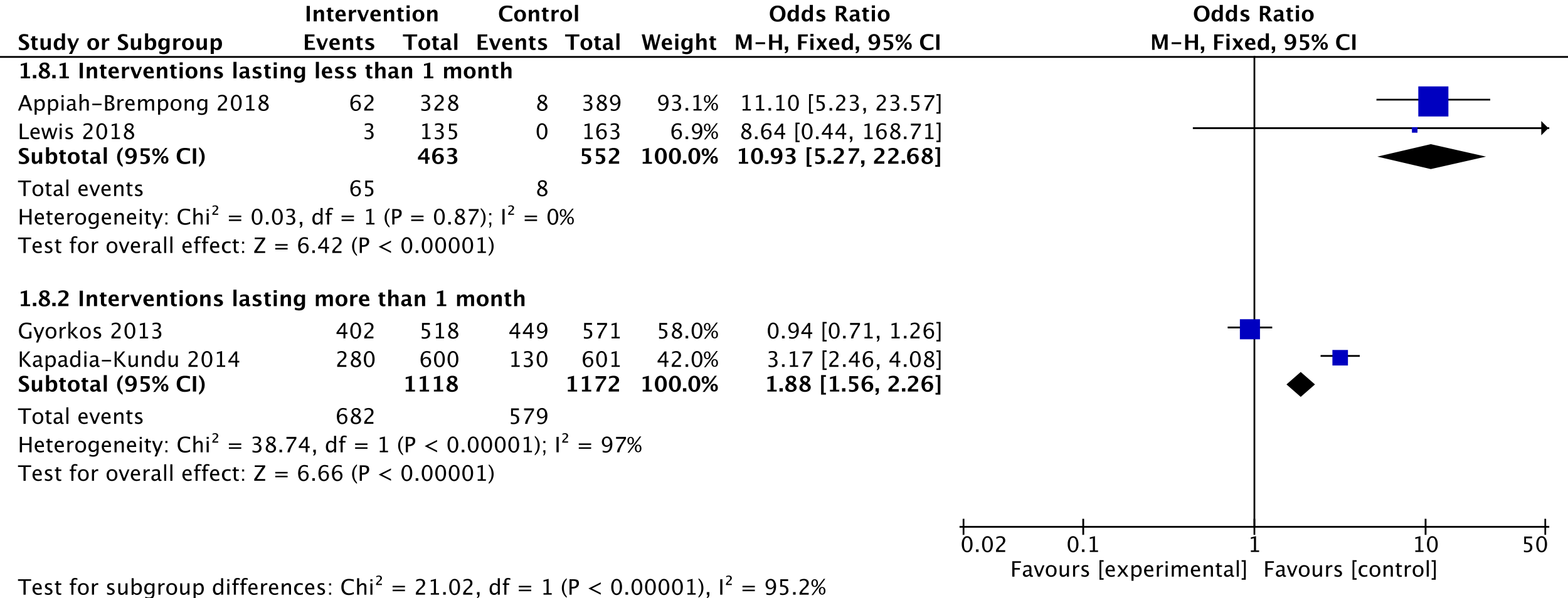


### Analysis 1.9. Handwashing with soap practices


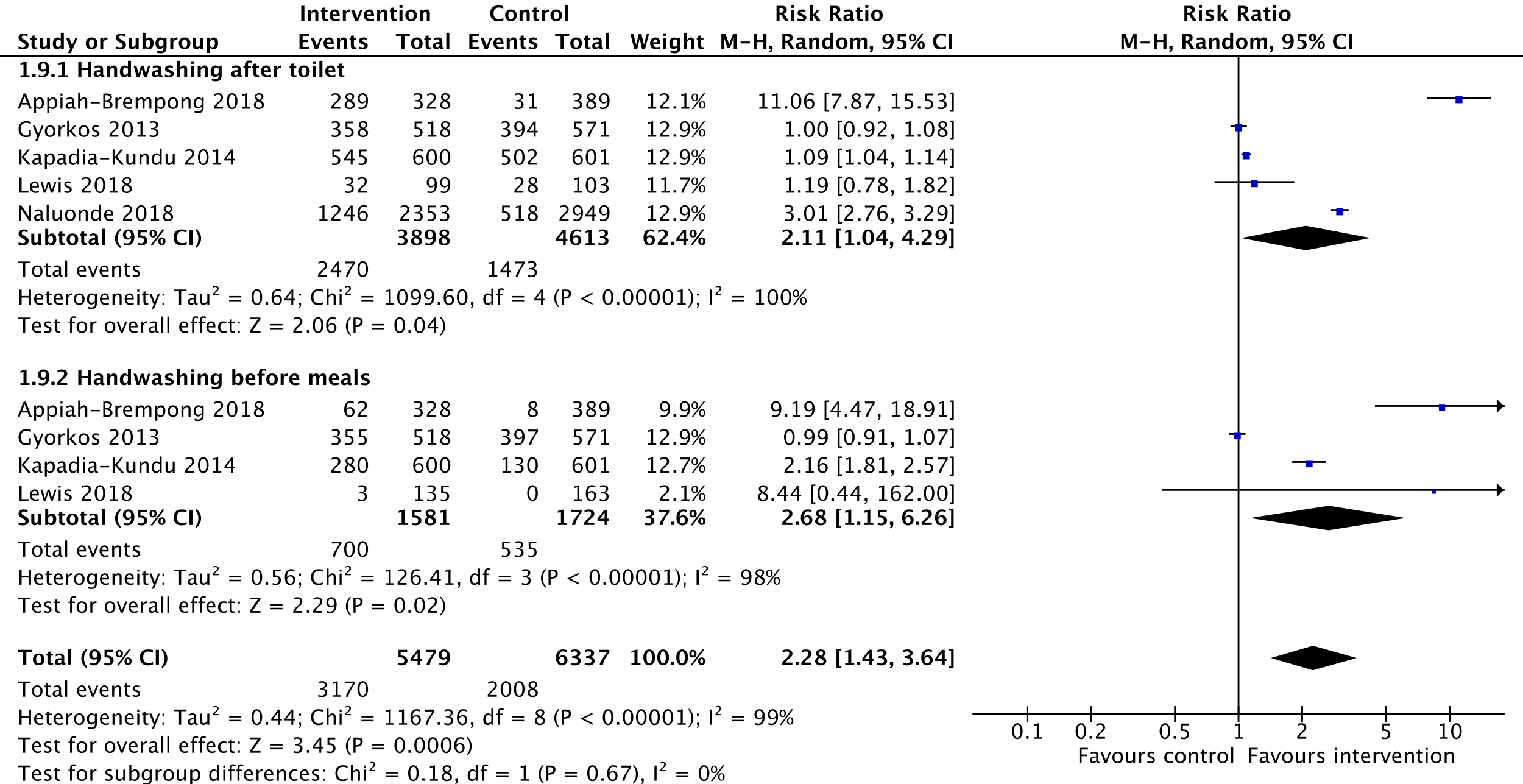


### Analysis 1.10. School absenteeism due to any infections


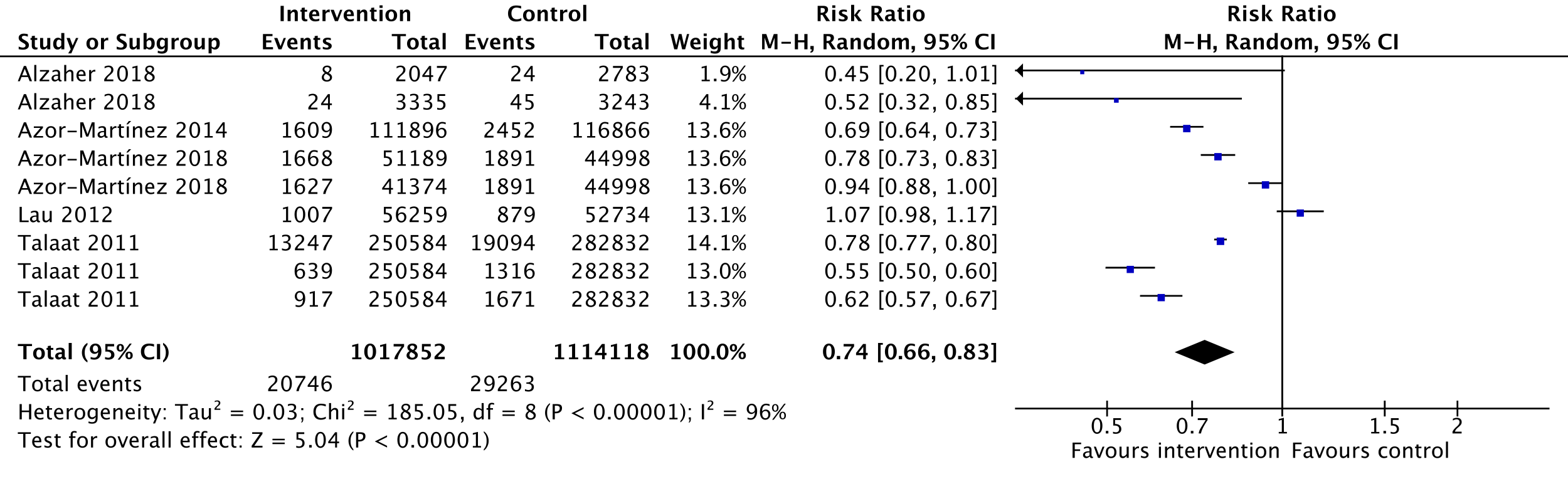


### Analysis 1.11. School absenteeism due to respiratory infections


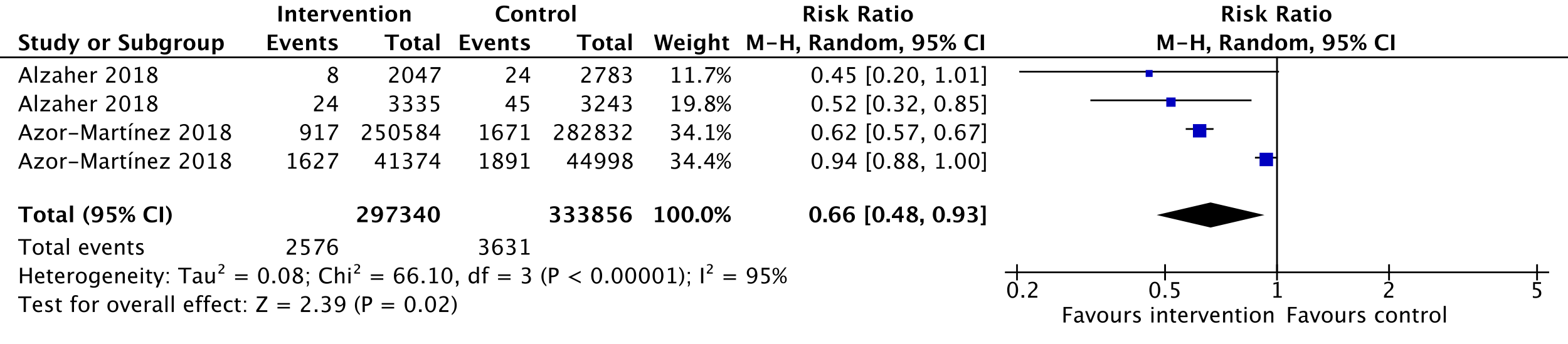


## Comparison 2: Genital hygiene interventions versus standard curriculum

### Analysis 2.1. Attitudes in genital hygiene


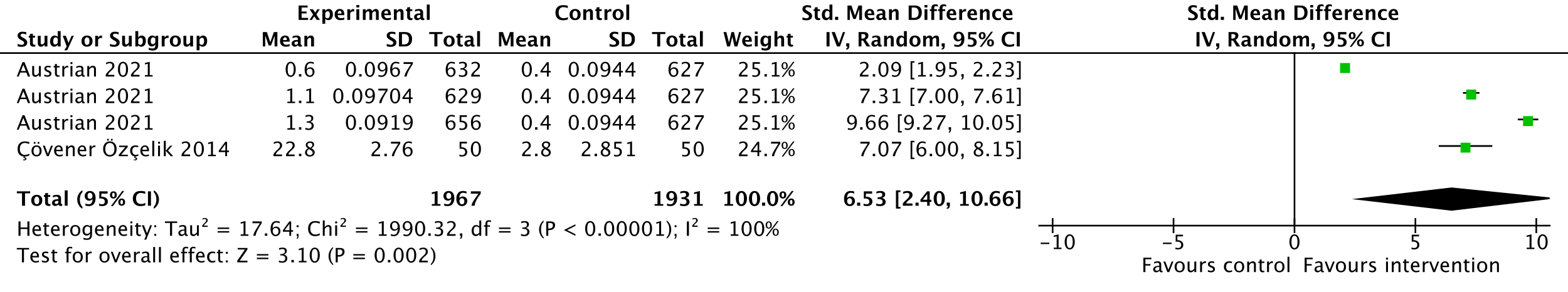


### Analysis 2.2. Practices in genital hygiene


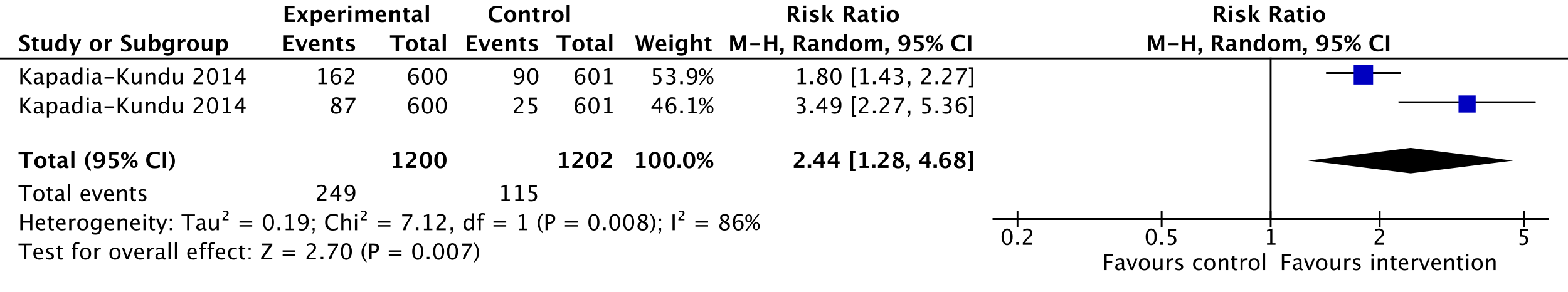


## Comparison 3: Oral hygiene interventions vs standard curriculum

### Analysis 3.1. Oral hygiene


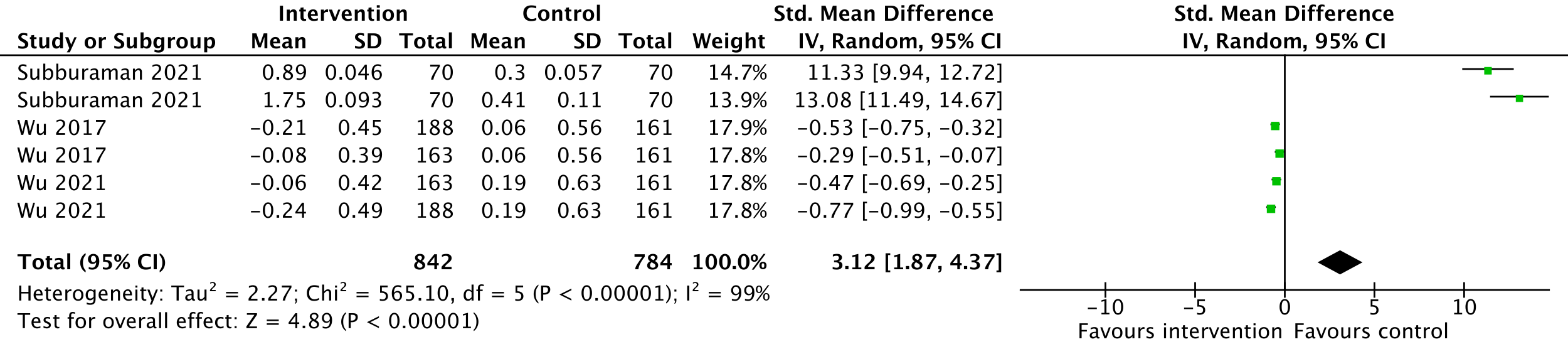


### Analysis 3.2. Subgroup analysis by study population for oral hygiene


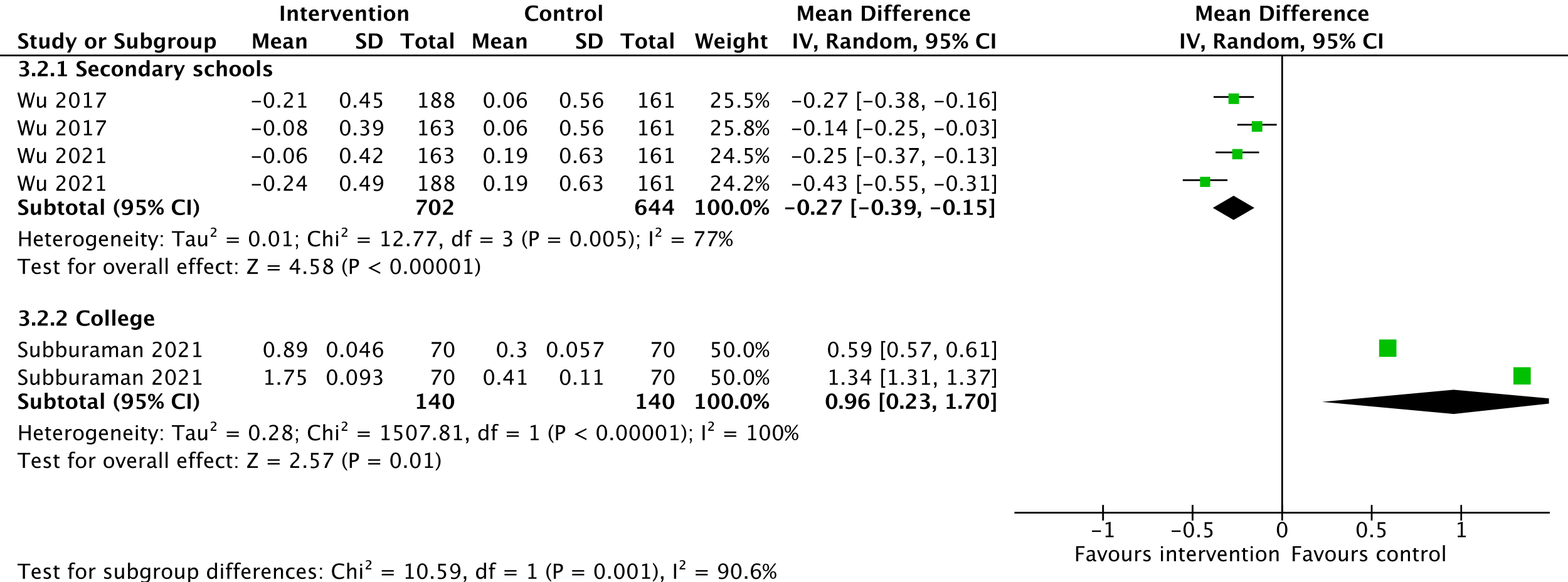


### Analysis 3.3. Dental status


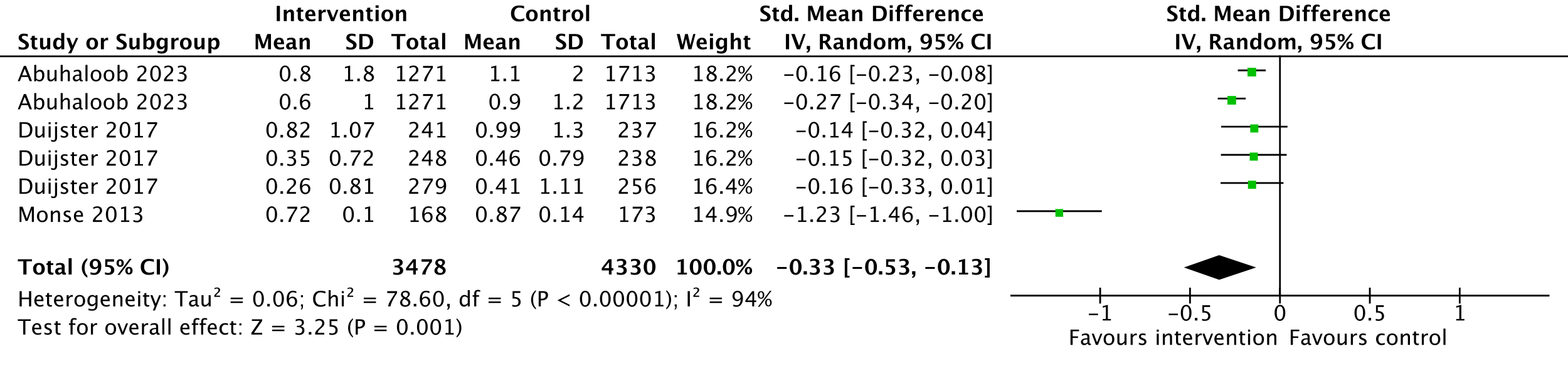


### Analysis 3.4. Dental caries


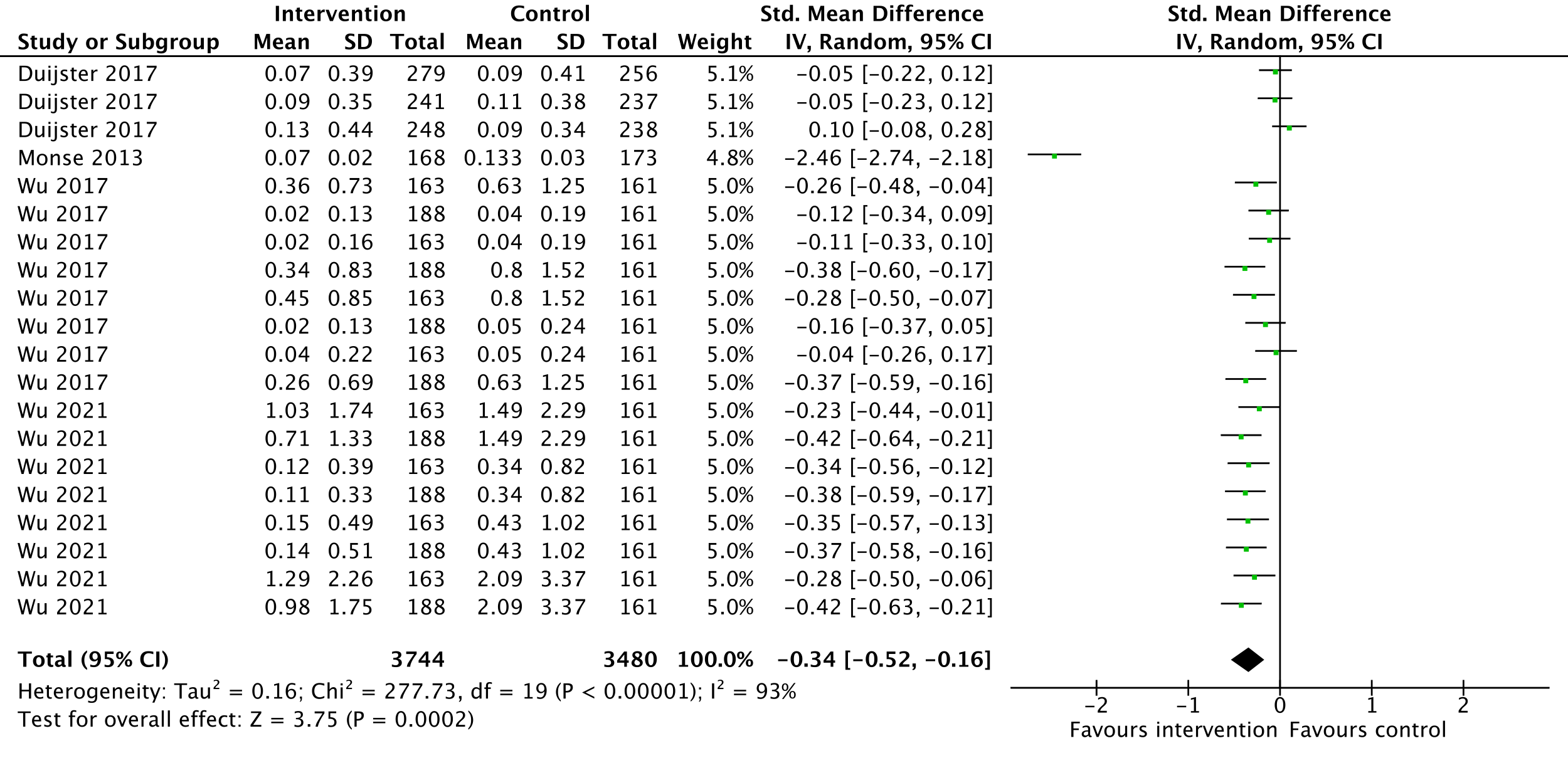

Supplement: S2 Appendix — (DOCX) [file pone.0308390.s005.docx]
