# Supplementary material for: The effects of school-based hygiene intervention programme: Systematic review and meta-analysis
Source: PLoS One. 2024 Oct 8;19(10):e0308390. doi: 10.1371/journal.pone.0308390 (PMC11460677; doi:10.1371/journal.pone.0308390)
Supplement: S3 Appendix — (DOCX) [file pone.0308390.s006.docx]

**HYGIENE SYSTEMATIC REVIEW**

**S3 Appendix Summary of risk of bias assessment of included studies**

**Overall summary of risk of bias assessment of included studies**

**
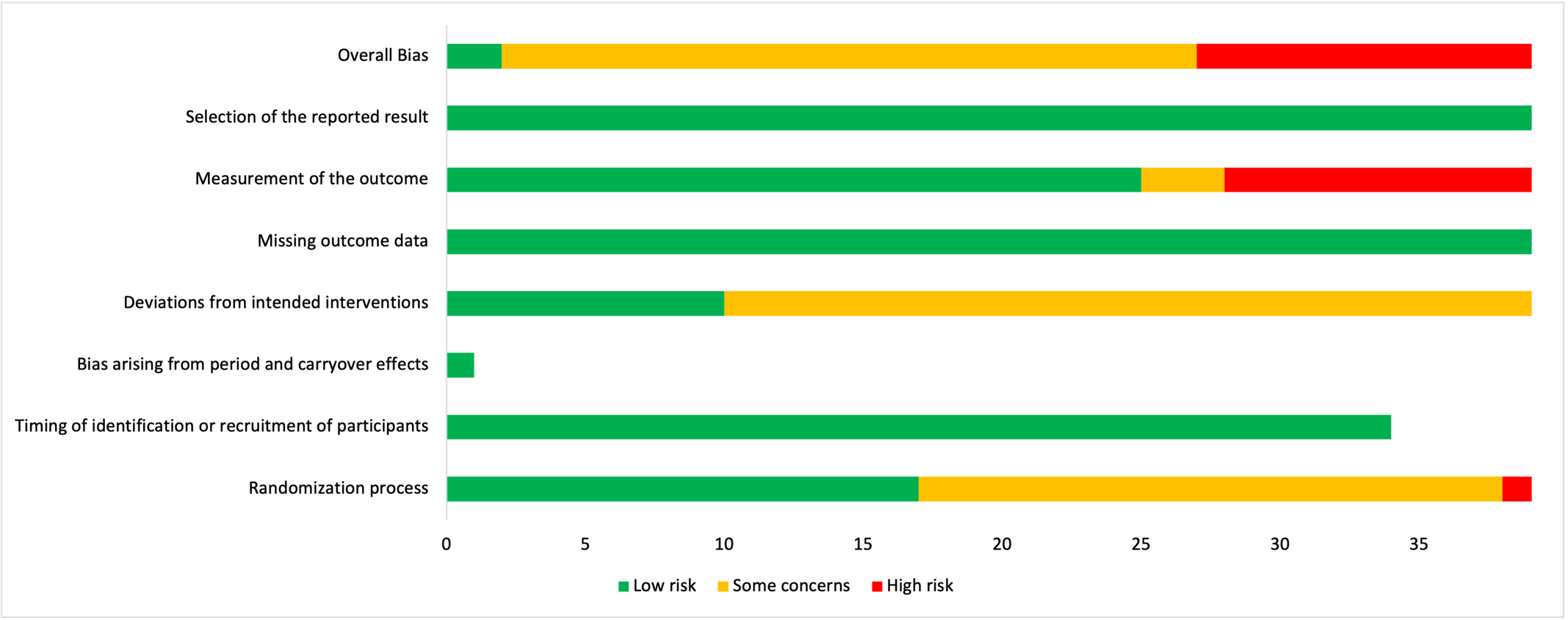
**

**Summary of risk of bias of cluster randomized controlled trials**

|  | **Randomization process** | **Timing of identification or recruitment of participants** | **Deviations from intended interventions** | **Missing outcome data** | **Measurement of the outcome** | **Selection of the reported result** | **Overall Bias** |
| --- | --- | --- | --- | --- | --- | --- | --- |
| Total number of studies = 34 |  |  |  |  |  |  |  |
| Low risk | 13 | 34 | 3 | 34 | 22 | 34 | 0 |
| Some concerns | 19 | 0 | 31 | 0 | 3 | 0 | 24 |
| High risk | 2 | 0 | 0 | 0 | 9 | 0 | 10 |

List of studies included under this category:

1. Abuhaloob 2023
2. Alzaher, 2018
3. Appiah-Brempong 2018
4. Aragie 2012
5. Austrian 2021
6. Azor-Martínez 2014
7. Azor-Martínez 2018
8. Bieri 2013
9. Biran, 2014
10. Bowen, 2007
11. Çövener Özçelik 2014
12. Duijster, 2017
13. Gyorkos, 2013
14. Huang 2021
15. Kapadia-kundu, 2014
16. Landsdown, 2002
17. Larsen 2021
18. Lau 2012
19. Lewis 2018
20. Maftuchan 2020
21. Makata 2021
22. Melo 2021
23. Monse, 2013
24. Naluonde, 2018
25. Parmar 2024
26. Öncu 2021
27. Patel 2012
28. Rosen, 2011
29. Ryom 2022
30. Shirzad 2016
31. Talaat 2011
32. Thériault 2014
33. Wu 2017
34. Wu 2021

*Detailed ROB assessment for these studies can be obtained from the authors.

**Summary of risk of bias of non-cluster randomized controlled trials**

|  | **Randomization process** | **Deviations from intended interventions** | **Missing outcome data** | **Measurement of the outcome** | **Selection of the reported result** | **Overall Bias** |
| --- | --- | --- | --- | --- | --- | --- |
| Total number of studies = 6 |  |  |  |  |  |  |
| Low risk | 3 | 6 | 6 | 2 | 65 | 2 |
| Some concerns | 2 | 0 | 0 | 0 | 0 | 0 |
| High risk | 1 | 0 | 0 | 4 | 0 | 4 |

List of studies included under this category:

1. Subburaman 2012
2. Almutairi 2023
3. Nastiti 2023
4. Ram Surath Kumar 2022
5. Sharma 2023
6. Pai Khot 2024

**Summary of risk of bias of crossover trials**

|  | **Randomization process** | **Bias arising from period and carryover effects** | **Deviations from intended interventions** | **Missing outcome data** | **Measurement of the outcome** | **Selection of the reported result** | **Overall Bias** |
| --- | --- | --- | --- | --- | --- | --- | --- |
| Total number of studies = 1 |  |  |  |  |  |  |  |
| Low risk | 1 | 1 | 1 | 1 | 1 | 0 | 0 |
| Some concerns | 0 | 0 | 0 | 0 | 0 | 1 | 1 |
| High risk | 0 | 0 | 0 | 0 | 0 | 0 | 0 |

List of studies included under this category:

1. Gerald 2012

**Risk of bias of non-cluster randomized controlled trials**

1. **Subburaman 2012**

| Reference | Subburaman N, Parangimalai DM, Iyer K, Sukumaran A. Effectiveness of social media based oral health promotion programme among 18-20 year old city college students - A comparative study. Indian J Dent Res. 2021 Oct-Dec;32(4):467-471. doi: 10.4103/ijdr.ijdr_1128_20. PMID: 35645073. |
| --- | --- |

| **Domain 1. Randomization process** | | |
| --- | --- | --- |
|  | Authors’ Judgement | Note |
| 1.1 Was the allocation sequence random? | Yes | A research collaborator who did not take part in the later stages of the study allocated all the participants into intervention and control groups, with a 1:1 ratio based on the coin toss method |
| 1.2 Was the allocation sequence concealed until participants were enrolled and assigned to interventions? | Yes |  |
| 1.3 Did baseline differences between intervention groups suggest a problem with the randomization process? | No |  |
| 1.0 Algorithm result | Low risk |  |
| 1.0 Assessor's judgement | Low risk |  |

| **Domain 2. Deviations from intended interventions** | | |
| --- | --- | --- |
|  | Authors’ Judgement | Note |
| 2.1 Were participants aware of their assigned intervention during the trial? | Yes | Participants were aware of the intervention received |
| 2.2 Were carers and people delivering the interventions aware of partcipants' assigned intervention during the trial? | Yes |  |
| 2.3 Were there deviations from the intended intervention that arose because of the trial context? | No information | Researchers delivering the intervention through WhatsApp were mostly likely aware of the assigned intervention group |
| 2.4 Were these deviations likely to have affected the outcome? | Not applicable |  |
| 2.5 Were these deviations from intended intervention balanced between groups | Not applicable |  |
| 2.6 Was an appropriate analysis used to estimate the effect of assignment to intervention? | Yes | ITT analysis was most likely applied |
| 2.7 Was there potential for a substantial impact of the failure to analyse participants in the group to which they were randomized? | Not applicable |  |
| 2.0 Algorithm result | Some concerns |  |
| 2.0 Assessor's Judgement | Low risk |  |

| **Domain 3. Missing outcome data** | | |
| --- | --- | --- |
|  | Authors’ Judgement | Note |
| 3.1 Were data for this outcome available for all, or nearly all, participants randomized? | Yes | Data for this outcome was available for all participants |
| 3.2 Is there evidence that the result was not biased by missing outcome data? | Not applicable |  |
| 3.3 Could missingness in the outcome depend on its true value? | Not applicable |  |
| 3.4 Is it likely that missingness in the outcome depended on its true value? | Not applicable |  |
| 3.0 Algorithm result | Low risk |  |
| 3.0 Assessor's judgement | Low risk |  |

| **Domain 4. Measurement of the Outcome** | | |
| --- | --- | --- |
|  | Authors’ Judgement | Note |
| 4.1 Was the method of measuring the outcome inappropriate? | No |  |
| 4.2 Could measurement or ascertainment of the outcome have differed between intervention groups? | No | Same measurement methods were used at the different time points |
| 4.3 Were outcome assessors aware of the intervention received by study participants? | Yes | It is not known if the assessors for the OHI-S and MGI scores had the knowledge of the assigned intervention. However, KAP was collected through a self-administered questionnaire |
| 4.4 Could assessment of the outcome have been influenced by knowledge of intervention received | Yes | It is likely that the outcomes could have been influenced by the knowledge of the intervention received as the outcomes were assessed through self-administered questionnaires |
| 4.5 Is it likely that assessment of the outcome was influenced by knowledge of intervention received? | Yes |  |
| 4.0 Algorithm result | High risk |  |
| 4.0 Assessor's judgement | High risk |  |

| **Domain 5. Selection of the reported result** | | |
| --- | --- | --- |
|  | Authors’ Judgement | Note |
| 5.1 Were the data that produced this result analysed in accordance with a pre-specified analysis plan that was finalised before unblinded outcome data were available for analysis? | Yes | Pre-specified intentions were described sufficiently in the article |
| 5.2 Is the numerical result being assessed likely to have been selected, on the basis of the results, from multiple eligible outcome measurements within the outcome domain? | No | The numerical results assessed were reported accordingly |
| 5.3 Is the numerical result being assessed likely to have been selected, on the basis of the results, from multiple eligible anlayses of the data? | No |  |
| 5.0 Algorithm result | Low risk |  |
| 5.0 Assessor's judgement | Low risk |  |

| **Domain 6. Overall Bias** | Algorithm's overall Judgement | High risk |
| --- | --- | --- |
|  | Assessor's overall Judgement | High risk |

1. **Almutairi 2023**

| Reference | Almutairi KS, Okmi EA, Alnofaiei SS, Alshamari WK, Almutairi SH, Alsuwailem SI, et al. The Effects of Health Education on the Awareness of Antimicrobial Resistance Among High School Students in Riyadh, Saudi Arabia During 2023: A Quasi-experimental Study. Cureus. 2023;15(7):e41639. Epub 2023/08/11. doi: 10.7759/cureus.41639. |
| --- | --- |

| **Domain 1. Randomization process** | | |
| --- | --- | --- |
|  | Authors’ Judgement | Note |
| 1.1 Was the allocation sequence random? | No | This was a quasi-experimental study |
| 1.2 Was the allocation sequence concealed until participants were enrolled and assigned to interventions? | Probably no |  |
| 1.3 Did baseline differences between intervention groups suggest a problem with the randomization process? | No |  |
| 1.0 Algorithm result | Some concerns |  |
| 1.0 Assessor's judgement | Some concerns |  |

| **Domain 2. Deviations from intended interventions** | | |
| --- | --- | --- |
|  | Authors’ Judgement | Note |
| 2.1 Were participants aware of their assigned intervention during the trial? | Probably yes |  |
| 2.2 Were carers and people delivering the interventions aware of partcipants' assigned intervention during the trial? | Yes |  |
| 2.3 Were there deviations from the intended intervention that arose because of the trial context? | No information |  |
| 2.4 Were these deviations likely to have affected the outcome? | Not applicable |  |
| 2.5 Were these deviations from intended intervention balanced between groups | Not applicable |  |
| 2.6 Was an appropriate analysis used to estimate the effect of assignment to intervention? | Yes |  |
| 2.7 Was there potential for a substantial impact of the failure to analyse participants in the group to which they were randomized? | Not applicable |  |
| 2.0 Algorithm result | Some concerns |  |
| 2.0 Assessor's Judgement | Low risk |  |

| **Domain 3. Missing outcome data** | | |
| --- | --- | --- |
|  | Authors’ Judgement | Note |
| 3.1 Were data for this outcome available for all, or nearly all, participants randomized? | Yes | Data for all outcomes were available for all participants |
| 3.2 Is there evidence that the result was not biased by missing outcome data? | Not applicable |  |
| 3.3 Could missingness in the outcome depend on its true value? | Not applicable |  |
| 3.4 Is it likely that missingness in the outcome depended on its true value? | Not applicable |  |
| 3.0 Algorithm result | Low risk |  |
| 3.0 Assessor's judgement | Low risk |  |

| **Domain 4. Measurement of the Outcome** | | |
| --- | --- | --- |
|  | Authors’ Judgement | Note |
| 4.1 Was the method of measuring the outcome inappropriate? | No |  |
| 4.2 Could measurement or ascertainment of the outcome have differed between intervention groups? | No | Same measurement methods were used at the different time points |
| 4.3 Were outcome assessors aware of the intervention received by study participants? | Yes | Participants completed a self-reported questionnaire |
| 4.4 Could assessment of the outcome have been influenced by knowledge of intervention received | Yes |  |
| 4.5 Is it likely that assessment of the outcome was influenced by knowledge of intervention received? | Yes |  |
| 4.0 Algorithm result | High risk |  |
| 4.0 Assessor's judgement | High risk |  |

| **Domain 5. Selection of the reported result** | | |
| --- | --- | --- |
|  | Authors’ Judgement | Note |
| 5.1 Were the data that produced this result analysed in accordance with a pre-specified analysis plan that was finalised before unblinded outcome data were available for analysis? | Yes | Pre-specified intentions were described sufficiently in the article |
| 5.2 Is the numerical result being assessed likely to have been selected, on the basis of the results, from multiple eligible outcome measurements within the outcome domain? | No | The numerical results assessed were reported accordingly |
| 5.3 Is the numerical result being assessed likely to have been selected, on the basis of the results, from multiple eligible anlayses of the data? | No |  |
| 5.0 Algorithm result | Low risk |  |
| 5.0 Assessor's judgement | Low risk |  |

| **Domain 6. Overall Bias** | Algorithm's overall Judgement | High risk |
| --- | --- | --- |
|  | Assessor's overall Judgement | High risk |

1. **Nastiti 2023**

| Reference | Nastiti AA, Triharini M, Pratiwi AH, Kamel Gouda AD. Educational intervention to improve menstrual hygiene management in adolescent girls in Kalimantan, Indonesia. J Pak Med Assoc. 2023;73(Suppl 2)(2):S13-s7. Epub 2023/04/25. doi: 10.47391/JPMA.Ind-S2-3. PubMed PMID: 37096693. |
| --- | --- |

| **Domain 1. Randomization process** | | |
| --- | --- | --- |
|  | Authors’ Judgement | Note |
| 1.1 Was the allocation sequence random? | No | This was a quasi-experimental study |
| 1.2 Was the allocation sequence concealed until participants were enrolled and assigned to interventions? | Probably no |  |
| 1.3 Did baseline differences between intervention groups suggest a problem with the randomization process? | No information |  |
| 1.0 Algorithm result | Some concerns |  |
| 1.0 Assessor's judgement | Some concerns |  |

| **Domain 2. Deviations from intended interventions** | | |
| --- | --- | --- |
|  | Authors’ Judgement | Note |
| 2.1 Were participants aware of their assigned intervention during the trial? | Probably yes | Participants were aware of the interventions received |
| 2.2 Were carers and people delivering the interventions aware of partcipants' assigned intervention during the trial? | Probably yes |  |
| 2.3 Were there deviations from the intended intervention that arose because of the trial context? | Not information |  |
| 2.4 Were these deviations likely to have affected the outcome? | Not applicable |  |
| 2.5 Were these deviations from intended intervention balanced between groups | Not applicable |  |
| 2.6 Was an appropriate analysis used to estimate the effect of assignment to intervention? | Yes | mITT was applied |
| 2.7 Was there potential for a substantial impact of the failure to analyse participants in the group to which they were randomized? | Not applicable |  |
| 2.0 Algorithm result | Some concerns |  |
| 2.0 Assessor's Judgement | Low risk |  |

| **Domain 3. Missing outcome data** | | |
| --- | --- | --- |
|  | Authors’ Judgement | Note |
| 3.1 Were data for this outcome available for all, or nearly all, participants randomized? | Yes | Data for all outcomes were available for all participants |
| 3.2 Is there evidence that the result was not biased by missing outcome data? | Not applicable |  |
| 3.3 Could missingness in the outcome depend on its true value? | Not applicable |  |
| 3.4 Is it likely that missingness in the outcome depended on its true value? | Not applicable |  |
| 3.0 Algorithm result | Low risk |  |
| 3.0 Assessor's judgement | Low risk |  |

| **Domain 4. Measurement of the Outcome** | | |
| --- | --- | --- |
|  | Authors’ Judgement | Note |
| 4.1 Was the method of measuring the outcome inappropriate? | No |  |
| 4.2 Could measurement or ascertainment of the outcome have differed between intervention groups? | No | Same measurement methods were used at the different time points |
| 4.3 Were outcome assessors aware of the intervention received by study participants? | Yes | Participants completed a self-reported questionnaire |
| 4.4 Could assessment of the outcome have been influenced by knowledge of intervention received | Yes |  |
| 4.5 Is it likely that assessment of the outcome was influenced by knowledge of intervention received? | Yes |  |
| 4.0 Algorithm result | High risk |  |
| 4.0 Assessor's judgement | High risk |  |

| **Domain 5. Selection of the reported result** | | |
| --- | --- | --- |
|  | Authors’ Judgement | Note |
| 5.1 Were the data that produced this result analysed in accordance with a pre-specified analysis plan that was finalised before unblinded outcome data were available for analysis? | Yes | Pre-specified intentions were described sufficiently in the article |
| 5.2 Is the numerical result being assessed likely to have been selected, on the basis of the results, from multiple eligible outcome measurements within the outcome domain? | No | The numerical results assessed were reported accordingly |
| 5.3 Is the numerical result being assessed likely to have been selected, on the basis of the results, from multiple eligible anlayses of the data? | No |  |
| 5.0 Algorithm result | Low risk |  |
| 5.0 Assessor's judgement | Low risk |  |

| **Domain 6. Overall Bias** | Algorithm's overall Judgement | High risk |
| --- | --- | --- |
|  | Assessor's overall Judgement | High risk |

1. **Ram Surath Kumar 2022**

| Reference | K RSK, Deshpande AP, Ankola AV, Sankeshwari RM, Jalihal S, Hampiholi V, Khot AJP, Hebbal M, Kotha SL, S LK. Effectiveness of a Visual Interactive Game on Oral Hygiene Knowledge, Practices, and Clinical Parameters among Adolescents: A Randomized Controlled Trial. Children (Basel). 2022 Nov 26;9(12):1828. doi: 10.3390/children9121828. |
| --- | --- |

| **Domain 1. Randomization process** | | |
| --- | --- | --- |
|  | Authors’ Judgement | Note |
| 1.1 Was the allocation sequence random? | Yes | Simple random sampling technique was employed by using a lottery method. / Allocation concealment was completed using the SNOSE (sequentially numbered, opaque, sealed enveloped) |
| 1.2 Was the allocation sequence concealed until participants were enrolled and assigned to interventions? | Yes |  |
| 1.3 Did baseline differences between intervention groups suggest a problem with the randomization process? | No |  |
| 1.0 Algorithm result | Low risk |  |
| 1.0 Assessor's judgement | Low risk |  |

| **Domain 2. Deviations from intended interventions** | | |
| --- | --- | --- |
|  | Authors’ Judgement | Note |
| 2.1 Were participants aware of their assigned intervention during the trial? | Probably yes | Participants were aware of the interventions received |
| 2.2 Were carers and people delivering the interventions aware of partcipants' assigned intervention during the trial? | Probably yes |  |
| 2.3 Were there deviations from the intended intervention that arose because of the trial context? | No information |  |
| 2.4 Were these deviations likely to have affected the outcome? | Not applicable |  |
| 2.5 Were these deviations from intended intervention balanced between groups | Not applicable |  |
| 2.6 Was an appropriate analysis used to estimate the effect of assignment to intervention? | Yes | mITT was applied |
| 2.7 Was there potential for a substantial impact of the failure to analyse participants in the group to which they were randomized? | Not applicable |  |
| 2.0 Algorithm result | Some concerns |  |
| 2.0 Assessor's Judgement | Low risk |  |

| **Domain 3. Missing outcome data** | | |
| --- | --- | --- |
|  | Authors’ Judgement | Note |
| 3.1 Were data for this outcome available for all, or nearly all, participants randomized? | Yes | Data for all outcomes were available for all participants |
| 3.2 Is there evidence that the result was not biased by missing outcome data? | Not applicable |  |
| 3.3 Could missingness in the outcome depend on its true value? | Not applicable |  |
| 3.4 Is it likely that missingness in the outcome depended on its true value? | Not applicable |  |
| 3.0 Algorithm result | Low risk |  |
| 3.0 Assessor's judgement | Low risk |  |

| **Domain 4. Measurement of the Outcome** | | |
| --- | --- | --- |
|  | Authors’ Judgement | Note |
| 4.1 Was the method of measuring the outcome inappropriate? | No |  |
| 4.2 Could measurement or ascertainment of the outcome have differed between intervention groups? | No | Same measurement methods were used at the different time points |
| 4.3 Were outcome assessors aware of the intervention received by study participants? | No | Clinical examinations were performed by investigators' bias was minimized by carrying out an examination by the same investigators who had been blinded by the grouping of children |
| 4.4 Could assessment of the outcome have been influenced by knowledge of intervention received | Not applicable |  |
| 4.5 Is it likely that assessment of the outcome was influenced by knowledge of intervention received? | Not applicable |  |
| 4.0 Algorithm result | Low risk |  |
| 4.0 Assessor's judgement | Low risk |  |

| **Domain 5. Selection of the reported result** | | |
| --- | --- | --- |
|  | Authors’ Judgement | Note |
| 5.1 Were the data that produced this result analysed in accordance with a pre-specified analysis plan that was finalised before unblinded outcome data were available for analysis? | Yes | Pre-specified intentions were described sufficiently in the article |
| 5.2 Is the numerical result being assessed likely to have been selected, on the basis of the results, from multiple eligible outcome measurements within the outcome domain? | No | The numerical results assessed were reported accordingly |
| 5.3 Is the numerical result being assessed likely to have been selected, on the basis of the results, from multiple eligible analyses of the data? | No |  |
| 5.0 Algorithm result | Low risk |  |
| 5.0 Assessor's judgement | Low risk |  |

| **Domain 6. Overall Bias** | Algorithm's overall Judgement | Some concerns |
| --- | --- | --- |
|  | Assessor's overall Judgement | Some concerns |

1. **Sharma 2023**

| Reference | Sharma M, Bachani R. Knowledge, Attitude, Practice, and Perceived Barriers for the Compliance of Standard Precautions among Medical and Nursing Students in Central India. International journal of environmental research and public health. 2023;20(8). Epub 2023/04/28. doi: 10.3390/ijerph20085487. |
| --- | --- |

| **Domain 1. Randomization process** | | |
| --- | --- | --- |
|  | Authors’ Judgement | Note |
| 1.1 Was the allocation sequence random? | No information | The only information about randomization methods is a statement that the study is randomized/ Allocation was made by school it is likely that the enrolling investigator had knowledge of the allocation |
| 1.2 Was the allocation sequence concealed until participants were enrolled and assigned to interventions? | No |  |
| 1.3 Did baseline differences between intervention groups suggest a problem with the randomization process? | No |  |
| 1.0 Algorithm result | High risk |  |
| 1.0 Assessor's judgement | High risk |  |

| **Domain 2. Deviations from intended interventions** | | |
| --- | --- | --- |
|  | Authors’ Judgement | Note |
| 2.1 Were participants aware of their assigned intervention during the trial? | Yes | Participants and people delivery interventions were aware of the interventions received |
| 2.2 Were carers and people delivering the interventions aware of partcipants' assigned intervention during the trial? | Yes |  |
| 2.3 Were there deviations from the intended intervention that arose because of the trial context? | No information |  |
| 2.4 Were these deviations likely to have affected the outcome? | Not applicable |  |
| 2.5 Were these deviations from intended intervention balanced between groups | Not applicable |  |
| 2.6 Was an appropriate analysis used to estimate the effect of assignment to intervention? | Yes | ITT was applied |
| 2.7 Was there potential for a substantial impact of the failure to analyse participants in the group to which they were randomized? | Not applicable |  |
| 2.0 Algorithm result | Some concerns |  |
| 2.0 Assessor's Judgement | Low risk |  |

| **Domain 3. Missing outcome data** | | |
| --- | --- | --- |
|  | Authors’ Judgement | Note |
| 3.1 Were data for this outcome available for all, or nearly all, participants randomized? | Yes | Data for all outcomes were available for all participants |
| 3.2 Is there evidence that the result was not biased by missing outcome data? | Not applicable |  |
| 3.3 Could missingness in the outcome depend on its true value? | Not applicable |  |
| 3.4 Is it likely that missingness in the outcome depended on its true value? | Not applicable |  |
| 3.0 Algorithm result | Low risk |  |
| 3.0 Assessor's judgement | Low risk |  |

| **Domain 4. Measurement of the Outcome** | | |
| --- | --- | --- |
|  | Authors’ Judgement | Note |
| 4.1 Was the method of measuring the outcome inappropriate? | No |  |
| 4.2 Could measurement or ascertainment of the outcome have differed between intervention groups? | No | Same measurement methods were used at the different time points |
| 4.3 Were outcome assessors aware of the intervention received by study participants? | Yes | Clinical examinations were performed by the principal investigator who also took part in delivering interventions |
| 4.4 Could assessment of the outcome have been influenced by knowledge of intervention received | Not applicable |  |
| 4.5 Is it likely that assessment of the outcome was influenced by knowledge of intervention received? | Not applicable |  |
| 4.0 Algorithm result | High risk |  |
| 4.0 Assessor's judgement | High risk |  |

| **Domain 5. Selection of the reported result** | | |
| --- | --- | --- |
|  | Authors’ Judgement | Note |
| 5.1 Were the data that produced this result analysed in accordance with a pre-specified analysis plan that was finalised before unblinded outcome data were available for analysis? | Yes | Pre-specified intentions were described sufficiently in the article |
| 5.2 Is the numerical result being assessed likely to have been selected, on the basis of the results, from multiple eligible outcome measurements within the outcome domain? | No | The numerical results assessed were reported accordingly |
| 5.3 Is the numerical result being assessed likely to have been selected, on the basis of the results, from multiple eligible anlayses of the data? | No |  |
| 5.0 Algorithm result | Low risk |  |
| 5.0 Assessor's judgement | Low risk |  |

| **Domain 6. Overall Bias** | Algorithm's overall Judgement | High risk |
| --- | --- | --- |
|  | Assessor's overall Judgement | High risk |

1. **Pai Khot 2024**

| Reference | Pai Khot AJ, Choudhury AR, Ankola AV, Sankeshwari RM, Hampiholi V, Hebbal M, et al. Evaluation of a “Picture Assisted Illustration Reinforcement” (PAIR) System for Oral Hygiene in Children with Autism: a Double-Blind Randomized Controlled Trial. Children. 2023;10(2). |
| --- | --- |

| **Domain 1. Randomization process** | | |
| --- | --- | --- |
|  | Authors’ Judgement | Note |
| 1.1 Was the allocation sequence random? | Yes | A simple random-sampling technique was employed by using the lottery method. Allocation concealment was administered using the SNOSE technique. |
| 1.2 Was the allocation sequence concealed until participants were enrolled and assigned to interventions? | Yes |  |
| 1.3 Did baseline differences between intervention groups suggest a problem with the randomization process? | No | There was no significant difference in baseline characteristics of ASQ, VSMS, ID and Frankl scale in children of both groups |
| 1.0 Algorithm result | Low risk |  |
| 1.0 Assessor's judgement | Low risk |  |

| **Domain 2. Deviations from intended interventions** | | |
| --- | --- | --- |
|  | Authors’ Judgement | Note |
| 2.1 Were participants aware of their assigned intervention during the trial? | Yes | Participants and carers were most likely aware of the intervention received |
| 2.2 Were carers and people delivering the interventions aware of partcipants' assigned intervention during the trial? | Yes |  |
| 2.3 Were there deviations from the intended intervention that arose because of the trial context? | No information |  |
| 2.4 Were these deviations likely to have affected the outcome? | Not applicable |  |
| 2.5 Were these deviations from intended intervention balanced between groups | Not applicable |  |
| 2.6 Was an appropriate analysis used to estimate the effect of assignment to intervention? | Yes | Results from all participants were analysed |
| 2.7 Was there potential for a substantial impact of the failure to analyse participants in the group to which they were randomized? | Not applicable |  |
| 2.0 Algorithm result | Some concerns |  |
| 2.0 Assessor's Judgement | Some concerns |  |

| **Domain 3. Missing outcome data** | | |
| --- | --- | --- |
|  | Authors’ Judgement | Note |
| 3.1 Were data for this outcome available for all, or nearly all, participants randomized? | Yes | Data for all outcomes were available for all participants |
| 3.2 Is there evidence that the result was not biased by missing outcome data? | Not applicable |  |
| 3.3 Could missingness in the outcome depend on its true value? | Not applicable |  |
| 3.4 Is it likely that missingness in the outcome depended on its true value? | Not applicable |  |
| 3.0 Algorithm result | Low risk |  |
| 3.0 Assessor's judgement | Low risk |  |

| **Domain 4. Measurement of the Outcome** | | |
| --- | --- | --- |
|  | Authors’ Judgement | Note |
| 4.1 Was the method of measuring the outcome inappropriate? | No |  |
| 4.2 Could measurement or ascertainment of the outcome have differed between intervention groups? | No | Same measurement methods were used at the different time points |
| 4.3 Were outcome assessors aware of the intervention received by study participants? | No | The type of intervention given by the investigator was masked from the examiners and was blinded from group assignment |
| 4.4 Could assessment of the outcome have been influenced by knowledge of intervention received | Not applicable |  |
| 4.5 Is it likely that assessment of the outcome was influenced by knowledge of intervention received? | Not applicable |  |
| 4.0 Algorithm result | Low risk |  |
| 4.0 Assessor's judgement | Low risk |  |

| **Domain 5. Selection of the reported result** | | |
| --- | --- | --- |
|  | Authors’ Judgement | Note |
| 5.1 Were the data that produced this result analysed in accordance with a pre-specified analysis plan that was finalised before unblinded outcome data were available for analysis? | Yes | Pre-specified intentions were described sufficiently in the article |
| 5.2 Is the numerical result being assessed likely to have been selected, on the basis of the results, from multiple eligible outcome measurements within the outcome domain? | No | The numerical results assessed were reported accordingly |
| 5.3 Is the numerical result being assessed likely to have been selected, on the basis of the results, from multiple eligible anlayses of the data? | No |  |
| 5.0 Algorithm result | Low risk |  |
| 5.0 Assessor's judgement | Low risk |  |

| **Domain 6. Overall Bias** | Algorithm's overall Judgement | Some concerns |
| --- | --- | --- |
|  | Assessor's overall Judgement | Low risk |

**Risk of bias of crossover trials**

1. **Gerald 2012**

| Reference | Gerald, L. B., Gerald, J. K., Zhang, B., McClure, L. A., Bailey, W. C., & Harrington, K. F. (2012). Can a school-based hand hygiene program reduce asthma exacerbations among elementary school children?. Journal of allergy and clinical immunology, 130(6), 1317-1324. |
| --- | --- |

| **Domain 1a. Randomization process** | | |
| --- | --- | --- |
|  | Authors’ Judgement | Note |
| 1.1 Was the allocation sequence random? | Yes | After being matched in terms of size and percentage of students eligible for free and reduced lunch, individual schools were randomized to receive usual care and then intervention (sequence 1) or intervention and then usual care (sequence 2). The project statistician generated the allocation sequence and assigned schools to sequence. The sequence was concealed until the intervention group was assigned. The sequence was concealed until the intervention group was assigned |
| 1.2 Was the allocation sequence concealed until clusters were enrolled and assigned to interventions? | Yes |  |
| 1.3 Did baseline differences between intervention groups suggest a problem with the randomization process? | No | Sequence 1 schools (61.8%) had a higher percentage of white students than sequence 2 schools (39.8%, P < .01); otherwise, all measured characteristics were similar across the 2 sequences. |
| 1.0 Algorithm result | Low Risk |  |
| 1.0 Assessor's judgement | Low Risk |  |

| **Domain S: Bias arising from period and carryover effects** | | |
| --- | --- | --- |
|  | Authors’ Judgement | Note |
| S.1 Was the number of participants allocated to each of the two sequences equal or nearly equal? | Yes | 233 students were assigned to Sequence 1 while 294 were assigned to Sequence 2. 16 were transferred to different sequences |
| S.2 Were period effects accounted for in the analysis? | NA |  |
| S.3 Was there sufficient time for any carryover effects to have dissapered before outcome assessment in the second period? | Yes | The 10-week summer break was thought to be of sufficient length to minimize this possibility |
| S.0 Algorithm result | Low Risk |  |
| S.0 Assessor's Judgement | Low Risk |  |

| **Domain 2. Bias due to deviations from intended interventions** | | |
| --- | --- | --- |
|  | Authors’ Judgement | Note |
| 2.1 Were participants aware of their assigned intervention during each period of the trial? | Yes | Children and school employee were not blinded to group assignment |
| 2.2 Were carers and people delivering the interventions aware of participants' assigned intervention during each period of the trial? | Yes | Written informed conset was obtained from parents, and written assent was obtained from the students. Children and school employee were not blinded to group assignment |
| 2.3 Were there deviations from the intended intervention that arose because of the trial context? / 2.3 Were important non-protocol-interventions balanced between interventions? | No information |  |
| 2.4 Were these deviations likely to have affected the outcome? / 2.4 Were there failures in implementing the intervention that could have affected the outcome? | NA |  |
| 2.5 Were these deviations from intended intervention balanced between interventions? / 2.5 Was there non-adherence to the assigned intervention regimen that could have affected participants' outcomes? | NA |  |
| 2.6 Was an appropriate analysis used to estimate the effect of assignment to intervention? / 2.6 Was an appropriate analysis used to estimate the effect of adhering to intervention? | Yes | All individuals were analysed according to the groups to which they were assigned |
| 2.7 Was there potential for substantial impact (on the result) of the failure to analyse participats in the group to which they were randomized? | NA |  |
| 2.0 Algorithm result | Some concerns |  |
| 2.0 Assessor's judgement | Some concerns |  |

| **Domain 3. Missing outcome data** | | |
| --- | --- | --- |
|  | Authors’ Judgement | Note |
| 3.1 Were data for this outcome available for all, or nearly all, participants randomized? | Yes | Data was available for both sequences |
| 3.2 Is there evidence that the result was not biased by missing outcome data? | NA |  |
| 3.3 Could missingness in the outcome depend on its true value? | NA |  |
| 3.4 Is it likely that missingness in the outcome depended on its true value? | NA |  |
| 3.0 Algorithm result | Low Risk |  |
| 3.0 Assessor's judgement | Low Risk |  |

| **Domain 4. Measurement of the Outcome** | | |
| --- | --- | --- |
|  | Authors’ Judgement | Note |
| 4.1 Was the method of measuring the outcome inappropriate? | No |  |
| 4.2 Could measurement or ascertainment of the outcome have differed between intervention groups? | No |  |
| 4.3 Were outcome assessors aware of the intervention received by study participants? | No | Investigators and study staff conducting telephone interviews were blinded |
| 4.4 Could assessment of the outcome have been influenced by knowledge of intervention received | NA |  |
| 4.5 Is it likely that assessment of the outcome was influenced by knowledge of intervention received? | NA |  |
| 4.0 Algorithm result | Low Risk |  |
| 4.0 Assessor's judgement | Low Risk |  |

| **Domain 5. Selection of the reported result** | | |
| --- | --- | --- |
|  | Authors’ Judgement | Note |
| 5.1 Were the data that produced this result analysed in accordance with a pre-specified analysis plan that was finalised before unblinded outcome data were available for analysis? | Yes | Pre-specified intentions were described sufficiently in the article |
| 5.2 Is the numerical result being assessed likely to have been selected, on the basis of the results, from multiple eligible outcome measurements within the outcome domain? | No | The numerical results assessed were reported accordingly |
| 5.3 Is the numerical result being assessed likely to have been selected, on the basis of the results, from multiple eligible analyses of the data? | No |  |
| 5.4 Is a result based on data from both periods sought, but unavailable on the basis of carryover having been identified? | No |  |
| 5.0 Algorithm result | Low Risk |  |
| 5.0 Assessor's judgement | Low Risk |  |

| **Domain 6. Overall Bias** | Algorithm's overall Judgement | Some concerns |
| --- | --- | --- |
|  | Assessor's overall Judgement | Some concerns |
